# Supplementary material for: Masking is good, but conforming is better: The consequences of masking non-conformity within the college classroom
Source: PLoS One. 2025 Jan 16;20(1):e0312392. doi: 10.1371/journal.pone.0312392 (PMC11737661; doi:10.1371/journal.pone.0312392)

**Supplemental Online Materials**

[**Study 1 5**](#_1pm6lspth4ah)

[**Study 1 Deviations from preregistration 5**](#_ctzzkeqmvko2)

[**Study 1 Participant Gender and Mask wearing 5**](#_5cmfmhhdp1pc)

[Fig S1.1. participant gender distributions by masking behavior 5](#_2u8vgo90l9gl)

[**Study 1: Impact of masking on gender perception 6**](#_r17z2of5bwt4)

[Inferring Gender from Classroom and Mask-Wearing Behavior. 6](#_1un9v5ypdijs)

[Fig S1.2. perceived target gender by target masking behavior. Error bars are SEs 7](#_ridrup0jpsr)

[Fig S1.3. perceived target gender by target and class masking behavior. Error bars are SEs 7](#_su03t9mfx76w)

[**Study 1 Models: Likeability 8**](#_txpk3hdunzxc)

[B’s gender unspecified; masking on Classroom Fit 8](#_4cwoivhvgk05)

[B’s gender specified; masking on Classroom Fit 8](#_imzp1ibi3za2)

[All data; masking on Classroom Fit 8](#_hnvreud3usc7)

[**Study 1 Summary: Other DVs. 9**](#_kgp23uvcnkn)

[**Study 1 Models: Other DVs (when target gender is unspecified) 10**](#_zbtufr5t7mgi)

[Threatening 10](#_4xv5hrv913b2)

[Warm/Caring 10](#_detbvyj2zoxx)

[Competence 11](#_5qvvu68p9bnw)

[Attractiveness 11](#_ddl004lpk1rj)

[Know-it-All 12](#_agxdegybv3b4)

[Emotional Strength 12](#_or6wxbqlbo6r)

[Independence 12](#_y78dnzlw1ell)

[Fear of loud sounds 13](#_4dn5rp1vlcvh)

[**Study 1 Models: target gender specified 14**](#_523ab6pawmyq)

[Threatened 14](#_o1ms2mc9zu3k)

[Warm/caring 14](#_ef1goneamnah)

[Competence 15](#_d8qqsyq498y9)

[Attractiveness 15](#_wvozuzrjrcik)

[Know-it-all 15](#_56gnnuflnb6w)

[Emotional strength 16](#_eicyahp31mw9)

[Independence 16](#_ivksmmaee7bw)

[Fear of loud sounds 17](#_dt3w5b7hr9xt)

[Study 1: Impact of Participant Gender on Perceptions. 17](#_5yq9rpa7bsyl)

[**Study 1: 4-way interaction models 19**](#_nlkhvhiovbry)

[Likeability 19](#_u6d806dp2nxq)

[**Study 2 21**](#_bs80s9wagwuw)

[**Study 2: Changes made to original preregistration prior to data collection 21**](#_eh5xkt8ui0rb)

[Study 2: How to read this section of the supplement 21](#_ps0iiihuvs1d)

[Study 2 Exclusions and inclusions Dependent Variables, Processing, and Exclusions 22](#_3sq7ml5i1q5v)

[Attention checks (these are exclusion criteria): 22](#_dgzztyxsejc3)

[Study 2 Final Participant Exclusions 22](#_9e03e1oup5pl)

[Study 2 Participants per cell (Classroom fit, must have min of 60) 23](#_f5asnn1qala8)

[Target Gender: 23](#_3o9ye5i4slou)

[Study 2 Distribution of Genders 23](#_8buuo8etfo2h)

[Study 2 Cronbach’s Alpha 23](#_no620e7ais4r)

[Classroom Fit 23](#_j0vxbeyhcm7z)

[Alpha 23](#_5cqykhvesrn0)

[Self Presentation Scale 23](#_ftxdyhlfyei5)

[Alpha 23](#_oobycfv51yss)

[Social Acceptance Scale 23](#_2ny9a0532q4y)

[Reverse code 23](#_7bl3fa94akyl)

[Alpha 24](#_997twqaye3mt)

[Social Contribution Scale 24](#_7o6870wb22j)

[Reverse code 24](#_y18ceap1a9k4)

[Alpha 24](#_e3y4v2nx7zu6)

[Participant characteristics 24](#_gpyyiafghk8f)

[Figure S2.1. Distribution of participant mask-wearing behavior 24](#_r6t2wr2wbwy4)

[Figure S2.2. Political orientation and masking. 25](#_txxajzr65mpi)

[Figure S2.3. Histogram of political orientation. 26](#_hymnl4avy6lg)

[3) Specific Analyses, by preregistered research question 26](#_2k9boeojxbbq)

[Question 1 26](#_5p2ba8hiwd4y)

[ClassFit ~ TargetMask*ClassMask (No Immune Status Info Given) 26](#_sxd3ssu1g67k)

[ClassFit ~ TargetMask*ClassMask (Full dataset) 26](#_y589v77pieo2)

[Tukey’s HSD No Immune Info Given 27](#_392a3zu51133)

[Tukey’s HSD Full Dataset 28](#_w0ehweei8341)

[Figures 29](#_u94w8fs68a6f)

[Figure S2.4. Bat chart of classroom fit by masking behavior and immune-status for Study 2. 29](#_ms5pvdcxdx7w)

[Question 2 29](#_knb6sim0jd52)

[Participant.Mask.Wearing ~ Participant.Gender (Full Dataset) 29](#_cv6mx8pff8ij)

[Perceived.Target.Gender ~ Target.Mask 30](#_46she8f4skx)

[Full Dataset 30](#_vwauki7jmq91)

[Immune Status Unknown 30](#_29ncrtbl3nxd)

[Target is immunocompromised 30](#_pl4otzxp1rxx)

[Classmate is immunocompromised 30](#_fbyzcch2jsh6)

[Professor is immunocompromised 30](#_wblaqzrfdymd)

[Perceived.Target.gender ~ Target.Mask * Class.Mask 31](#_j0u1qet2hl2p)

[Perceived.Target.gender ~ Target.Mask * Class.Mask (Full Dataset) 31](#_q2las18ul3fr)

[Perceived.Target.gender and masking: Tukey HSD (Full Dataset) 31](#_cro12fk7nctz)

[Perceived.Target.gender and masking: Figure (Full Dataset) 33](#_d8ib15fgs4ez)

[Figure S2.5. Bar chart of perceived gender by masking behavior for Study 2. 33](#_4097665aww81)

[Perceived.Target.gender ~ Target.Mask * Class.Mask (Immune Status Unknown) 33](#_kgdi8zufgppz)

[Perceived.Target.gender and masking: Figure (No Immune Info Known) 34](#_uatjhlefa27a)

[Figure S2.6. Bar chart of perceived gender by masking behavior for only individuals who received no immune-status info for Study 2. 34](#_mxha5z3sv4jq)

[Question 3 34](#_2mag7tbk1yrv)

[Perceived.Target.Gender ~ Immune.Status.Condition 35](#_wzmwmzv2bxru)

[Perceived.Target.Gender ~ Target.Mask * Class.Mask * Immunity.Status 35](#_7ptjrk9lnozo)

[Question 4 36](#_88ubgvi1q3jl)

[SelfPresentation ~ TargetMask*ClassMask (Full Dataset) 36](#_pdqcky9hf1te)

[SelfPresentation TukeyHSD (Full Dataset) 37](#_51pe55fel773)

[Self Presentation Effect Sizes (Full Dataset) 37](#_rjfx72fnthki)

[SocialContributuion ~ TargetMask*ClassMask (Full Dataset) 38](#_bnmd038te28f)

[Social Contribution Tukey’s (Full Dataset) 38](#_g7nw1ryvxipl)

[Social Contribution effect Sizes (Full Dataset) 38](#_qd0lqksj8q6s)

[SocialAcceptance ~ TargetMask*ClassMask (Full Dataset) 40](#_xamobeevruna)

[SocialAcceptance Tukey’s (Full Dataset) 40](#_7ttompe9hxqq)

[SocialAcceptance effect Sizes (Full Dataset) 40](#_40mifwh17iwm)

[Question 5 42](#_oruy6rodctzf)

[ClassroomFit ~ Target.Mask * Class.Mask * Immunity.Status 42](#_3jlcdn2hs1e8)

[Only Tukey-HSD that was sig: Unmasked Conformers (Classroom Fit) 44](#_mwm6njxvvtz8)

[Oneway Analysis of Classroom Fit By Immunocompromised MaskClassification=Masked Conformer 45](#_o48bu0w880r4)

[Oneway Analysis of Classroom Fit By Immunocompromised MaskClassification=Masked Deviant 45](#_r4j16r81qe09)

[Oneway Analysis of Classroom Fit By Immunocompromised MaskClassification=Unmasked Conformer 46](#_h5qcpyq73uao)

[Oneway Analysis of Classroom Fit By Immunocompromised MaskClassification=Unmasked Deviant 47](#_nwa8nqrv61ea)

[SelfPresentation ~ Target.Mask * Class.Mask * Immunity.Status 47](#_gk4yjolsp5po)

[Social Contribution ~ Target.Mask * Class.Mask * Immunity.Status 48](#_c0zuvcsadi7j)

[Social Acceptance ~ Target.Mask * Class.Mask * Immunity.Status 48](#_nvpem7k5ejq4)

[Tukey’s HSD self Presentation 49](#_2l6tw4cqwqap)

[Tukey’s HSD Social Contribution 51](#_q5c53tkfq281)

[Tukey’s HSD Social Acceptance 53](#_7y4hsboiopzh)

[Exploratory Analyses 55](#_sgvwjzyata1q)

[Post-hoc correlation of Classroom Fit by Self Presentation & Masking 56](#_tc48dklsjqk7)

[Figure S2.7. Line chart of Classroom Fit and Self Presentation, by masking status 56](#_bb2d71niqznb)

[Secondary DVs 56](#_v8t8ujrr29j4)

[Social Acceptance. 56](#_y7ck829ftjm7)

[Social Contribution. 57](#_nfvzgppss4jd)

[Exploratory Analyses: impact of participant masking behavior on the effect of mask conformity 57](#_1ro1o74iv2de)

[Classroom Fit 57](#_kzz1bjpl5r4s)

[Self Presentation 57](#_salwdsd7tdn2)

[Social Contribution 57](#_sthtvlyp3k6l)

[Social Acceptance 58](#_nlcho0lvah5j)

[**Study 3 59**](#_e7tu8axhtnbh)

[Study 3 Inclusion Screening. 59](#_hynkilknkhxy)

[**Study 3: Demographics 60**](#_wl9whaylf323)

[Race 60](#_99truymx8xm3)

[Minoritized Gender 61](#_4j42rbb1x2yi)

[Participant Immune Status 61](#_vrvxqigbdb23)

[Participant Masking Behavior 62](#_mk8il98g15hw)

[Study 3: Correlation of Actual and Preferred Masking Behavior 62](#_x0y9cxysu938)

[Study 3: post-hoc analyses of demographics and masking 63](#_3c2yvalcybnz)

[Participant Masking Preference 63](#_kv3crjethqg5)

[Masking Preference vs Behavior 64](#_y3pkah3vj4b0)

[Study 3: Classroom Enrollment Data 64](#_wl2as4isyp7t)

[Study 3: Effects of Demographics on DVs 64](#_l1zxk0yewn65)

#

# Study 1

## Study 1 Deviations from preregistration

All deviations from the pre-registration are clearly labeled as such. We made a terminological change, renaming the “Likeability” scale to the “Classroom Fit” scale. We believe this new name better captures the contents of the scale. The components of these two scales do not differ from one another -- they are the same thing.

## Study 1 Participant Gender and Mask wearing
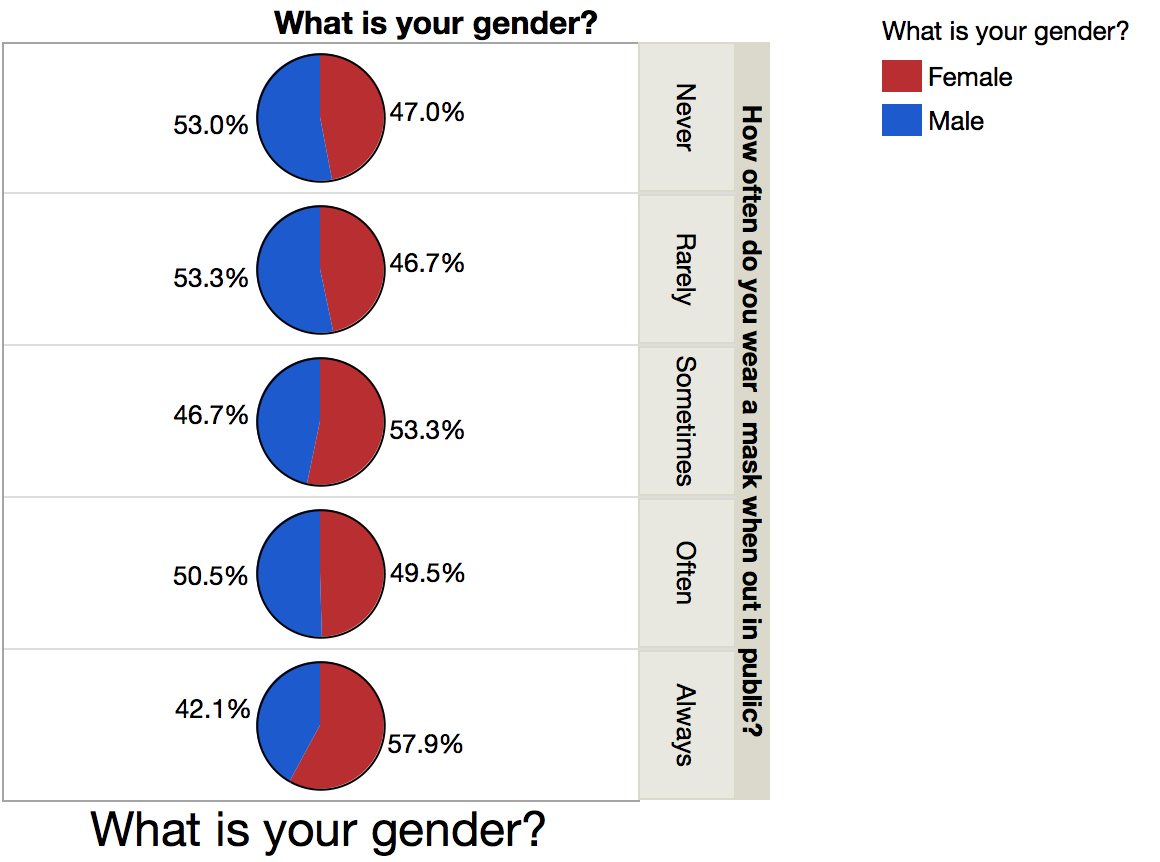


### Fig S1.1. participant gender distributions by masking behavior

## Study 1: Impact of masking on gender perception

### Inferring Gender from Classroom and Mask-Wearing Behavior.

We first consider the *N* = 451 participants who did not learn B’s gender. For them, inferences about the target’s gender could only be based on the class B was in, the class’s behavior, and B’s mask wearing behavior. Overall, one-sample Wilcoxon signed rank analyses revealed that B was assumed to be male (*M* = .28, *p*<.0001; *d =* .59), consistent with the notion that men are more likely to take engineering courses. However, there was a significant effect of mask-wearing behavior, such that individuals who always wore a mask (*M* = .233) were less likely to be assumed to be male (*t*(445) = 2.05, *p* = .041, *d* = .19) than those who never wore a mask (*M* = .33). These data confirm some previously-reported observation that masking behavior is more typical for women than for men ([Bainbridge, Allsopp, & Pollet, preprint](https://psyarxiv.com/njv9a/); [Chuang & Liu, 2020](https://ideas.repec.org/a/ebl/ecbull/eb-20-00882.html); [Haischer et al., 2020](https://journals.plos.org/plosone/article?id=10.1371/journal.pone.0240785); [Haischer et al., 2022](https://www.medrxiv.org/content/10.1101/2022.01.18.22269479v1.full-text); [Hearne & Niño, 2021](https://link.springer.com/article/10.1007/s40615-020-00941-1)), an observation that matches the participant effects described above. However, in planned analyses predicting perceived gender from the target’s masking behavior, the class’s masking behavior, and their interaction, the effect of target gender (*B* = -.04, *SE* = .02, *p* = .052) no longer appeared, and instead there were effects of class masking behavior (*B* = .057, *SE* = .02, *p* = .01), such that B was more likely to be perceived to be a man when the class always masked (see Figures S1.2 and S1.3).


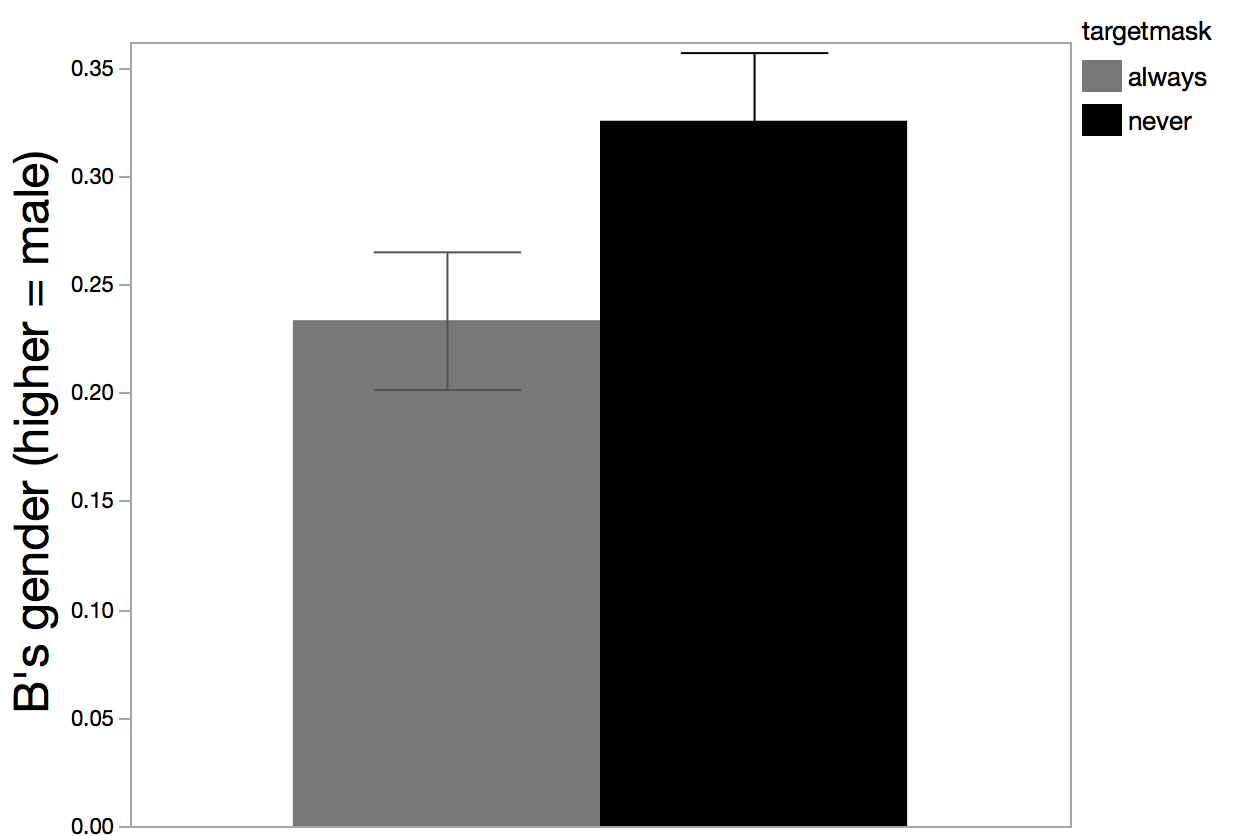


### Fig S1.2. perceived target gender by target masking behavior. Error bars are SEs


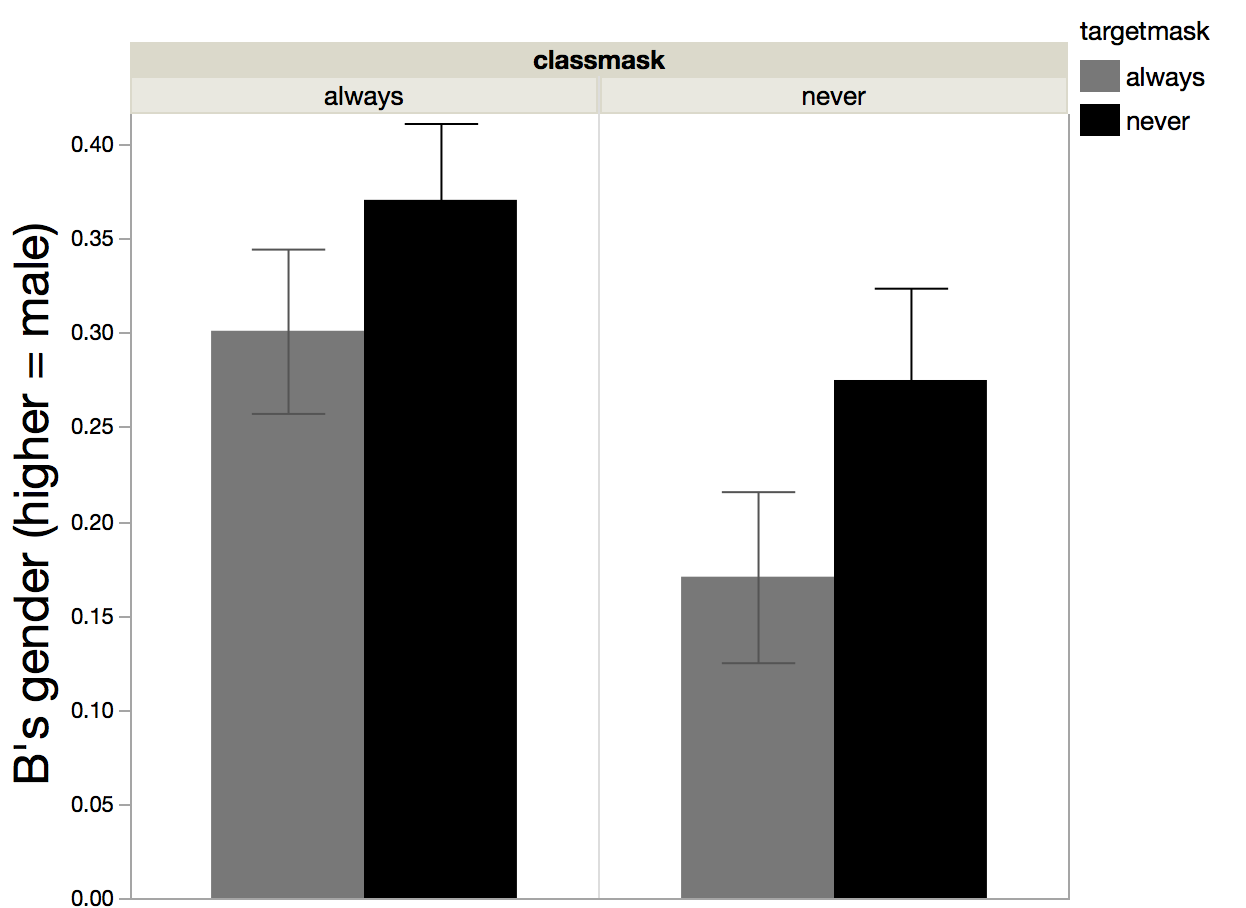


### Fig S1.3. perceived target gender by target and class masking behavior. Error bars are SEs

## Study 1 Models: Likeability

### B’s gender unspecified; masking on Classroom Fit

| **Term** | **Estimate** | **Std Error** | **t Ratio** | **Prob>\|t\|** |
| --- | --- | --- | --- | --- |
| Intercept | 0.1623149 | 0.01342 | 12.09 | <.0001* |
| targetmask[always] | 0.1126047 | 0.01342 | 8.39 | <.0001* |
| classmask[always] | -0.051269 | 0.01342 | -3.82 | 0.0002* |
| targetmask[always]*classmask[always] | 0.2465364 | 0.01342 | 18.37 | <.0001* |

### B’s gender specified; masking on Classroom Fit

| Term | Estimate | Std Error | t Ratio | Prob>\|t\| |
| --- | --- | --- | --- | --- |
| Intercept | 0.165233 | 0.008559 | 19.30 | <.0001* |
| targetgender[man] | 0.009858 | 0.008559 | 1.15 | 0.2497 |
| targetmask[always] | 0.058352 | 0.008559 | 6.82 | <.0001* |
| targetgender[man]*targetmask[always] | 0.0030748 | 0.008559 | 0.36 | 0.7195 |
| classmask[always] | -0.051496 | 0.008559 | -6.02 | <.0001* |
| targetgender[man]*classmask[always] | -0.006554 | 0.008559 | -0.77 | 0.4440 |
| targetmask[always]*classmask[always] | 0.2288331 | 0.008559 | 26.74 | <.0001* |
| targetgender[man]*targetmask[always]*classmask[always] | -0.004431 | 0.008559 | -0.52 | 0.6048 |

##

### All data; masking on Classroom Fit

| *Term* | *Estimate* | *Std Error* | *t Ratio* | *Prob>\|t\|* |
| --- | --- | --- | --- | --- |
| *Intercept* | *0.1644255* | *0.007273* | *22.61* | *<.0001** |
| *targetmask[always]* | *0.0770891* | *0.007273* | *10.60* | *<.0001** |
| *classmask[always]* | *-0.05213* | *0.007273* | *-7.17* | *<.0001** |
| *targetmask[always]*classmask[always]* | *0.2347948* | *0.007273* | *32.28* | *<.0001** |

## Study 1 Summary: Other DVs.

There were interactions between the target’s and class’s mask-wearing behaviors for most items. These interactions were generally best described as a conformity effect: those who conformed to the mask-wearing behavior displayed by their classmates were deemed more attractive, to be less of a know-it-all, and to be less independent than their non-conforming peers. There was also an overall effect of conformity for how threatening B was perceived to be, though the highest level of perceived threat was when B *never* wore a mask but their classmates *always* did.

Some of these conformity effects were impacted by B’s gender. A three-way interaction of masking behavior and gender on perceptions of independence revealed that men who conformed to the masking-behavior of their classmates were perceived as significantly less independent than women who did the same. And, there was a main effect of gender on perceptions of attractiveness, such that women were rated as more attractive than men. This effect was additive with the effect of mask-wearing-conformity, meaning that women who conformed to the mask-wearing behaviors of their peers were deemed most attractive.

Not all interactions indicated simple conformity effects. Specifically, those who always wore a mask were perceived as more warm/caring, and more competent than those who never did, although this effect was largest when individuals in the classroom typically wore masks. In addition, those who always wore a mask were assumed to be more afraid of loud noises (our proxy for overall fearfulness). However, this effect was largest when their classmates never wore masks, and this effect was additive with a main effect of gender, such that women who wore masks when their classmates did not were perceived to be most likely to be afraid of loud sounds.

## Study 1 Models: Other DVs (when target gender is unspecified)

### Threatening

| **Term** | **Estimate** | **Std Error** | **t Ratio** | **Prob>\|t\|** |
| --- | --- | --- | --- | --- |
| Intercept | -0.359643 | 0.019328 | -18.61 | <.0001* |
| targetmask[always] | -0.15835 | 0.019328 | -8.19 | <.0001* |
| classmask[always] | 0.127972 | 0.019328 | 6.62 | <.0001* |
| targetmask[always]*classmask[always] | -0.209792 | 0.019328 | -10.85 | <.0001* |

| Level |  |  |  | Mean |
| --- | --- | --- | --- | --- |
| never always | A |  |  | 0.1364706 |
| always never |  | B |  | -0.4361739 |
| never never |  | B | C | -0.5390566 |
| always always |  |  | C | -0.5998131 |

##

### Warm/Caring

| **Term** | **Estimate** | **Std Error** | **t Ratio** | **Prob>\|t\|** |
| --- | --- | --- | --- | --- |
| Intercept | 0.1795404 | 0.019216 | 9.34 | <.0001* |
| targetmask[always] | 0.1960447 | 0.019216 | 10.20 | <.0001* |
| classmask[always] | -0.046171 | 0.019216 | -2.40 | 0.0167* |
| targetmask[always]*classmask[always] | 0.1074085 | 0.019216 | 5.59 | <.0001* |

| Level |  |  |  | Mean |
| --- | --- | --- | --- | --- |
| always always | A |  |  | 0.4368224 |
| always never | A |  |  | 0.3143478 |
| never never |  | B |  | 0.1370755 |
| never always |  |  | C | -0.1700840 |

### Competence

| **Term** | **Estimate** | **Std Error** | **t Ratio** | **Prob>\|t\|** |
| --- | --- | --- | --- | --- |
| Intercept | 0.3824378 | 0.019016 | 20.11 | <.0001* |
| targetmask[always] | 0.1145898 | 0.019016 | 6.03 | <.0001* |
| classmask[always] | -0.033532 | 0.019016 | -1.76 | 0.0785 |
| targetmask[always]*classmask[always] | 0.042299 | 0.019016 | 2.22 | 0.0266* |

| Level |  |  |  | Mean |
| --- | --- | --- | --- | --- |
| always always | A |  |  | 0.50579439 |
| always never | A |  |  | 0.48826087 |
| never never |  | B |  | 0.34367925 |
| never always |  |  | C | 0.19201681 |

### Attractiveness

| **Term** | **Estimate** | **Std Error** | **t Ratio** | **Prob>\|t\|** |
| --- | --- | --- | --- | --- |
| Intercept | 0.121777 | 0.018016 | 6.76 | <.0001* |
| targetmask[always] | 0.0382812 | 0.018016 | 2.12 | 0.0342* |
| classmask[always] | 0.0004291 | 0.018016 | 0.02 | 0.9810 |
| targetmask[always]*classmask[always] | 0.0649333 | 0.018016 | 3.60 | 0.0003* |

##

| Level |  |  | Mean |
| --- | --- | --- | --- |
| always always | A |  | 0.22542056 |
| never never | A | B | 0.14800000 |
| always never | A | B | 0.09469565 |
| never always |  | B | 0.01899160 |

### Know-it-All

| **Term** | **Estimate** | **Std Error** | **t Ratio** | **Prob>\|t\|** |
| --- | --- | --- | --- | --- |
| Intercept | -0.016094 | 0.020074 | -0.80 | 0.4231 |
| targetmask[always] | -0.100159 | 0.020074 | -4.99 | <.0001* |
| classmask[always] | 0.0620507 | 0.020074 | 3.09 | 0.0021* |
| targetmask[always]*classmask[always] | -0.146826 | 0.020074 | -7.31 | <.0001* |

##

| Level |  |  |  | Mean |
| --- | --- | --- | --- | --- |
| never always | A |  |  | 0.2929412 |
| always never |  | B |  | -0.0314783 |
| never never |  | B | C | -0.1248113 |
| always always |  |  | C | -0.2010280 |

### Emotional Strength

| **Term** | **Estimate** | **Std Error** | **t Ratio** | **Prob>\|t\|** |
| --- | --- | --- | --- | --- |
| Intercept | 0.2200353 | 0.022578 | 9.75 | <.0001* |
| targetmask[always] | 0.0183332 | 0.022578 | 0.81 | 0.4172 |
| classmask[always] | -0.008472 | 0.022578 | -0.38 | 0.7077 |
| targetmask[always]*classmask[always] | -0.010551 | 0.022578 | -0.47 | 0.6405 |

| Level |  | Mean |
| --- | --- | --- |
| always never | A | 0.25739130 |
| always always | A | 0.21934579 |
| never always | A | 0.20378151 |
| never never | A | 0.19962264 |

### Independence

| **Term** | **Estimate** | **Std Error** | **t Ratio** | **Prob>\|t\|** |
| --- | --- | --- | --- | --- |
| Intercept | 0.3771714 | 0.022104 | 17.06 | <.0001* |
| targetmask[always] | 0.0160789 | 0.022104 | 0.73 | 0.4674 |
| classmask[always] | -0.012481 | 0.022104 | -0.56 | 0.5726 |
| targetmask[always]*classmask[always] | -0.123573 | 0.022104 | -5.59 | <.0001* |

| Level |  |  | Mean |
| --- | --- | --- | --- |
| always never | A |  | 0.52930435 |
| never always | A |  | 0.47218487 |
| always always |  | B | 0.25719626 |
| never never |  | B | 0.25000000 |

### Fear of loud sounds

| Term | Estimate | Std Error | t Ratio | Prob>\|t\| |
| --- | --- | --- | --- | --- |
| Intercept | -0.295806 | 0.019918 | -14.85 | <.0001* |
| targetmask[always] | 0.0710962 | 0.019918 | 3.57 | 0.0004* |
| classmask[always] | -0.049467 | 0.019918 | -2.48 | 0.0134* |
| targetmask[always]*classmask[always] | -0.051243 | 0.019918 | -2.57 | 0.0104* |

| Level |  |  | Mean |
| --- | --- | --- | --- |
| always never | A |  | -0.1240000 |
| always always |  | B | -0.3254206 |
| never always |  | B | -0.3651261 |
| never never |  | B | -0.3686792 |

## Study 1 Models: target gender specified

### Threatened

| **Term** | **Estimate** | **Std Error** | **t Ratio** | **Prob>\|t\|** |
| --- | --- | --- | --- | --- |
| Intercept | -0.362764 | 0.013668 | -26.54 | <.0001* |
| targetgender[man] | -0.008591 | 0.013668 | -0.63 | 0.5298 |
| targetmask[always] | -0.110179 | 0.013668 | -8.06 | <.0001* |
| targetgender[man]*targetmask[always] | -0.006681 | 0.013668 | -0.49 | 0.6251 |
| classmask[always] | 0.0986519 | 0.013668 | 7.22 | <.0001* |
| targetgender[man]*classmask[always] | 0.0080748 | 0.013668 | 0.59 | 0.5548 |
| targetmask[always]*classmask[always] | -0.143256 | 0.013668 | -10.48 | <.0001* |
| targetgender[man]*targetmask[always]*classmask[always] | -0.00433 | 0.013668 | -0.32 | 0.7515 |

| **Level** |  |  | **Mean** |
| --- | --- | --- | --- |
| never, always | A |  | -0.0107240 |
| always, never |  | B | -0.4298661 |
| never, never |  | B | -0.4942986 |
| always, always |  | B | -0.5178199 |

### Warm/caring

| **Term** | **Estimate** | **Std Error** | **t Ratio** | **Prob>\|t\|** |
| --- | --- | --- | --- | --- |
| Intercept | 0.2126241 | 0.012092 | 17.58 | <.0001* |
| targetgender[man] | -0.007458 | 0.012092 | -0.62 | 0.5376 |
| targetmask[always] | 0.1440898 | 0.012092 | 11.92 | <.0001* |
| targetgender[man]*targetmask[always] | 0.0108453 | 0.012092 | 0.90 | 0.3700 |
| classmask[always] | -0.064718 | 0.012092 | -5.35 | <.0001* |
| targetgender[man]*classmask[always] | -0.007806 | 0.012092 | -0.65 | 0.5188 |
| targetmask[always]*classmask[always] | 0.083278 | 0.012092 | 6.89 | <.0001* |
| targetgender[man]*targetmask[always]*classmask[always] | -0.015116 | 0.012092 | -1.25 | 0.2116 |

| **Level** |  |  |  | **Mean** |
| --- | --- | --- | --- | --- |
| always, always | A |  |  | 0.3749057 |
| always, never | A |  |  | 0.3402679 |
| never, never |  | B |  | 0.2168778 |
| never, always |  |  | C | -0.0794118 |

### Competence

| **Term** | **Estimate** | **Std Error** | **t Ratio** | **Prob>\|t\|** |
| --- | --- | --- | --- | --- |
| Intercept | 0.4213033 | 0.013353 | 31.55 | <.0001* |
| targetgender[man] | -0.008103 | 0.013353 | -0.61 | 0.5441 |
| targetmask[always] | 0.0808269 | 0.013353 | 6.05 | <.0001* |
| targetgender[man]*targetmask[always] | -0.014426 | 0.013353 | -1.08 | 0.2803 |
| classmask[always] | -0.035924 | 0.013353 | -2.69 | 0.0073* |
| targetgender[man]*classmask[always] | 0.0132543 | 0.013353 | 0.99 | 0.3212 |
| targetmask[always]*classmask[always] | 0.0355086 | 0.013353 | 2.66 | 0.0080* |
| targetgender[man]*targetmask[always]*classmask[always] | -0.019106 | 0.013353 | -1.43 | 0.1528 |

| **Level** |  |  | **Mean** |
| --- | --- | --- | --- |
| always, never | A |  | 0.50120536 |
| always, always | A |  | 0.50117925 |
| never, never | A |  | 0.41226244 |
| never, always |  | B | 0.26886878 |

### Attractiveness

| **Term** | **Estimate** | **Std Error** | **t Ratio** | **Prob>\|t\|** |
| --- | --- | --- | --- | --- |
| Intercept | 0.1715139 | 0.011514 | 14.90 | <.0001* |
| targetgender[man] | -0.052435 | 0.011514 | -4.55 | <.0001* |
| targetmask[always] | 0.0122242 | 0.011514 | 1.06 | 0.2887 |
| targetgender[man]*targetmask[always] | 0.0118531 | 0.011514 | 1.03 | 0.3035 |
| classmask[always] | -0.003318 | 0.011514 | -0.29 | 0.7733 |
| targetgender[man]*classmask[always] | -0.009601 | 0.011514 | -0.83 | 0.4046 |
| targetmask[always]*classmask[always] | 0.0353423 | 0.011514 | 3.07 | 0.0022* |
| targetgender[man]*targetmask[always]*classmask[always] | -0.012987 | 0.011514 | -1.13 | 0.2596 |

| **Level** |  |  | **Mean** |
| --- | --- | --- | --- |
| always, always | A |  | 0.21426540 |
| never, never | A | B | 0.19886878 |
| always, never | A | B | 0.15026786 |
| never, always |  | B | 0.12090498 |

### Know-it-all

| **Term** | **Estimate** | **Std Error** | **t Ratio** | **Prob>\|t\|** |
| --- | --- | --- | --- | --- |
| Intercept | -0.059949 | 0.013411 | -4.47 | <.0001* |
| targetgender[man] | 0.0170686 | 0.013411 | 1.27 | 0.2035 |
| targetmask[always] | -0.090635 | 0.013411 | -6.76 | <.0001* |
| targetgender[man]*targetmask[always] | -0.013866 | 0.013411 | -1.03 | 0.3015 |
| classmask[always] | 0.0636042 | 0.013411 | 4.74 | <.0001* |
| targetgender[man]*classmask[always] | -0.016109 | 0.013411 | -1.20 | 0.2300 |
| targetmask[always]*classmask[always] | -0.09619 | 0.013411 | -7.17 | <.0001* |
| targetgender[man]*targetmask[always]*classmask[always] | 0.0186693 | 0.013411 | 1.39 | 0.1643 |

| **Level** |  |  | **Mean** |
| --- | --- | --- | --- |
| never, always | A |  | 0.1904977 |
| always, never |  | B | -0.1179464 |
| never, never |  | B | -0.1300000 |
| always, always |  | B | -0.1830332 |

### Emotional strength

| **Term** | **Estimate** | **Std Error** | **t Ratio** | **Prob>\|t\|** |
| --- | --- | --- | --- | --- |
| Intercept | 0.2873755 | 0.014051 | 20.45 | <.0001* |
| targetgender[man] | -0.007444 | 0.014051 | -0.53 | 0.5964 |
| targetmask[always] | 0.0390667 | 0.014051 | 2.78 | 0.0055* |
| targetgender[man]*targetmask[always] | 0.0247874 | 0.014051 | 1.76 | 0.0781 |
| classmask[always] | -0.004593 | 0.014051 | -0.33 | 0.7439 |
| targetgender[man]*classmask[always] | -0.007516 | 0.014051 | -0.53 | 0.5928 |
| targetmask[always]*classmask[always] | -0.01883 | 0.014051 | -1.34 | 0.1806 |
| targetgender[man]*targetmask[always]*classmask[always] | -0.012384 | 0.014051 | -0.88 | 0.3784 |

| **Level** |  |  | **Mean** |
| --- | --- | --- | --- |
| always, never | A |  | 0.35285714 |
| always, always | A | B | 0.30297170 |
| never, always | A | B | 0.26266968 |
| never, never |  | B | 0.23440909 |

### Independence

| **Term** | **Estimate** | **Std Error** | **t Ratio** | **Prob>\|t\|** |
| --- | --- | --- | --- | --- |
| Intercept | 0.3958152 | 0.014574 | 27.16 | <.0001* |
| targetgender[man] | -0.023249 | 0.014574 | -1.60 | 0.1110 |
| targetmask[always] | -0.008115 | 0.014574 | -0.56 | 0.5778 |
| targetgender[man]*targetmask[always] | -0.014339 | 0.014574 | -0.98 | 0.3254 |
| classmask[always] | -0.005648 | 0.014574 | -0.39 | 0.6985 |
| targetgender[man]*classmask[always] | 2.0139e-5 | 0.014574 | 0.00 | 0.9989 |
| targetmask[always]*classmask[always] | -0.102644 | 0.014574 | -7.04 | <.0001* |
| targetgender[man]*targetmask[always]*classmask[always] | -0.0336 | 0.014574 | -2.31 | 0.0214* |

| **Level** |  |  | **Mean** |
| --- | --- | --- | --- |
| never, always | A |  | 0.50081448 |
| always, never | A |  | 0.49566964 |
| never, never |  | B | 0.30751131 |
| always, always |  | B | 0.27806604 |

### Fear of loud sounds

| **Term** | **Estimate** | **Std Error** | **t Ratio** | **Prob>\|t\|** |
| --- | --- | --- | --- | --- |
| Intercept | -0.320391 | 0.012955 | -24.73 | <.0001* |
| targetgender[man] | -0.037888 | 0.012955 | -2.92 | 0.0035* |
| targetmask[always] | 0.0761853 | 0.012955 | 5.88 | <.0001* |
| targetgender[man]*targetmask[always] | 0.012324 | 0.012955 | 0.95 | 0.3417 |
| classmask[always] | -0.02668 | 0.012955 | -2.06 | 0.0398* |
| targetgender[man]*classmask[always] | 0.0116273 | 0.012955 | 0.90 | 0.3697 |
| targetmask[always]*classmask[always] | -0.025462 | 0.012955 | -1.97 | 0.0497* |
| targetgender[man]*targetmask[always]*classmask[always] | 0.0038032 | 0.012955 | 0.29 | 0.7692 |

| **Level** |  |  |  | **Mean** |
| --- | --- | --- | --- | --- |
| always, never | A |  |  | -0.1953571 |
| always, always |  | B |  | -0.2965877 |
| never, never |  |  | C | -0.3945701 |
| never, always |  |  | C | -0.3976018 |

## **Study 1: Impact of Participant Gender on Perceptions**.

As preregistered, we assessed whether participants’ gender impacted perceptions of the target. As noted in our preregistration, these analyses were planned (in the sense that we believed that they were of theoretical interest and would be generative for future studies and theory-building) but exploratory (in the sense that we did not have specific theoretical predictions, and we knew that these analyses would only include a subset of our full dataset). As such, we believe these findings should be interpreted with caution, and primarily be used for motivating future work. These analyses only included the *N* = 863 participants who specified their binary gender *and* who learned B’s gender; there were 47-63 observations per cell.

Once again, as preregistered, we focus on the Classroom Fit scale (see SOM for analyses for all secondary DVs). We predicted Classroom Fit from target gender, participant gender, target mask-wearing, class mask-wearing, and all interactions. Full model reporting is available in SOM. We found a four-way interaction of target gender, participant gender, target masking, and class masking on Classroom Fit (*B* = .02, *SE* = .009, *p* = .033, η_p_^2^ = .005, see Fig. 2). Based on preliminary data visualizations (Fig. S1.4), we determined that the most effective way to break down (and interpret) this 4-way interaction would be to split our data by classroom masking behavior. Specifically, in classrooms where students *always* masked, there was only a main effect of mask conformity: B was rated as fitting in better when they conformed to the dominant masking behavior than when they deviated from it (*B* = .29, *SE* = .01, *p*<.0001, *d* = 2.13, [CI*_d_ =* 1.89-2.37]). However, in classrooms where students typically *never* masked, a three-way interaction of B’s masking behavior, B’s gender, and the participant’s gender emerged (*B* = -.02, *SE*  = .01, *p*= .037, η_p_^2^ = .01). Critically, *post-hoc* calculations of effect size (which we used because the post-hoc nature of these fine-grained analyses would lead to anti-conservative statistical tests) were consistent with the possibility that, in the classroom where students typically never masked, male participants rated mask-deviant women as fitting in worse than mask-deviant men (*d* = .47, [CI*_d_ =* .09-.85]); however, female participants did not rate mask-deviant women and mask-deviant men differently (*d* = .06, [CI*_d_ =* -.31 - .43]). In addition, men’s ratings of mask-deviant women were substantially lower than women’s ratings of mask-deviant women (*d* = .50 [CI*_d_ =* .11-.89]; see Fig. 2). Once again, men’s ratings of mask-deviant men did not differ from women’s ratings of mask-deviant men (*d* = .03 [CI*_d_ =* -.33 - .39]).


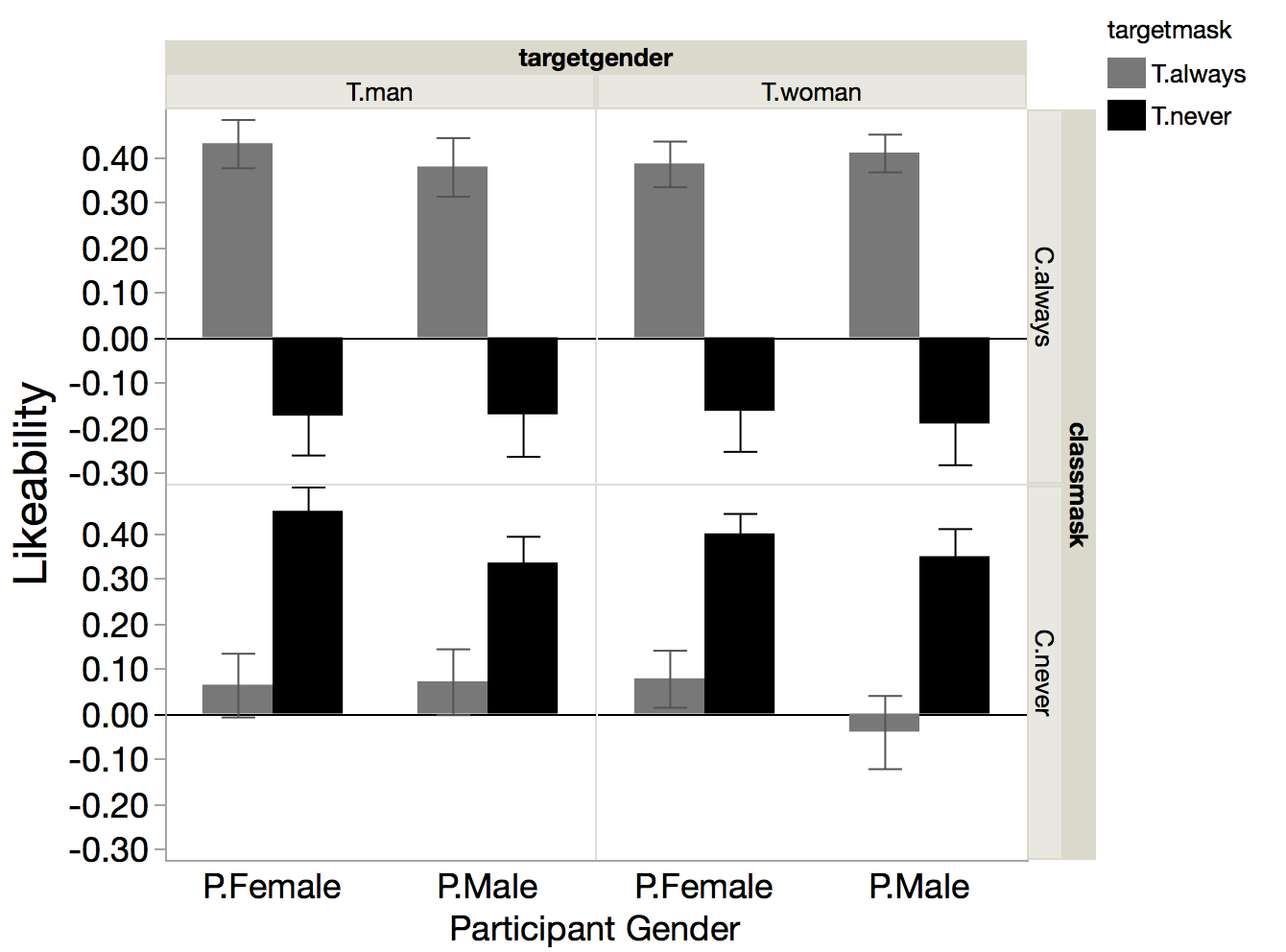


Figure S1.4. Classroom Fit by participant gender (lower x-axis), target gender (upper x-axis), class masking-status (right-hand y-axis) and target masking behavior (always = gray; never = black). Error bars are 95% CIs.

## Study 1: 4-way interaction models

### Likeability

| **Term** | **Estimate** | **Std Error** | **t Ratio** | **Prob>\|t\|** |
| --- | --- | --- | --- | --- |
| Intercept | 0.1634995 | 0.008686 | 18.82 | <.0001* |
| targetgender[T.man] | 0.0098382 | 0.008686 | 1.13 | 0.2577 |
| targetmask[T.always] | 0.0590583 | 0.008686 | 6.80 | <.0001* |
| targetgender[T.man]*targetmask[T.always] | 0.0041209 | 0.008686 | 0.47 | 0.6353 |
| classmask[C.always] | -0.050057 | 0.008686 | -5.76 | <.0001* |
| targetgender[T.man]*classmask[C.always] | -0.006883 | 0.008686 | -0.79 | 0.4283 |
| targetmask[T.always]*classmask[C.always] | 0.2292159 | 0.008686 | 26.39 | <.0001* |
| targetgender[T.man]*targetmask[T.always]*classmask[C.always] | -0.003573 | 0.008686 | -0.41 | 0.6809 |
| What is your gender?[P.Female] | 0.02058 | 0.008686 | 2.37 | 0.0180* |
| targetgender[T.man]*What is your gender?[P.Female] | -0.001069 | 0.008686 | -0.12 | 0.9021 |
| targetmask[T.always]*What is your gender?[P.Female] | -0.00328 | 0.008686 | -0.38 | 0.7058 |
| targetgender[T.man]*targetmask[T.always]*What is your gender?[P.Female] | -0.005178 | 0.008686 | -0.60 | 0.5512 |
| classmask[C.always]*What is your gender?[P.Female] | -0.013963 | 0.008686 | -1.61 | 0.1083 |
| targetgender[T.man]*classmask[C.always]*What is your gender?[P.Female] | 0.0066428 | 0.008686 | 0.76 | 0.4446 |
| targetmask[T.always]*classmask[C.always]*What is your gender?[P.Female] | 0.0035247 | 0.008686 | 0.41 | 0.6850 |
| targetgender[T.man]*targetmask[T.always]*classmask[C.always]*What is your gender?[P.Female] | 0.0185685 | 0.008686 | 2.14 | 0.0328* |

3-way breakdown

always mask

| **Term** | **Estimate** | **Std Error** | **t Ratio** | **Prob>\|t\|** |
| --- | --- | --- | --- | --- |
| Intercept | 0.1134421 | 0.013185 | 8.60 | <.0001* |
| targetgender[T.man] | 0.0029555 | 0.013185 | 0.22 | 0.8228 |
| targetmask[T.always] | 0.2882743 | 0.013185 | 21.86 | <.0001* |
| targetgender[T.man]*targetmask[T.always] | 0.000548 | 0.013185 | 0.04 | 0.9669 |
| What is your gender?[P.Female] | 0.0066172 | 0.013185 | 0.50 | 0.6160 |
| targetgender[T.man]*What is your gender?[P.Female] | 0.0055736 | 0.013185 | 0.42 | 0.6727 |
| targetmask[T.always]*What is your gender?[P.Female] | 0.0002449 | 0.013185 | 0.02 | 0.9852 |
| targetgender[T.man]*targetmask[T.always]*What is your gender?[P.Female] | 0.0133902 | 0.013185 | 1.02 | 0.3104 |

never mask

| **Term** | **Estimate** | **Std Error** | **t Ratio** | **Prob>\|t\|** |
| --- | --- | --- | --- | --- |
| Intercept | 0.2135569 | 0.011373 | 18.78 | <.0001* |
| targetgender[T.man] | 0.0167209 | 0.011373 | 1.47 | 0.1422 |
| targetmask[T.always] | -0.170158 | 0.011373 | -14.96 | <.0001* |
| targetgender[T.man]*targetmask[T.always] | 0.0076939 | 0.011373 | 0.68 | 0.4991 |
| What is your gender?[P.Female] | 0.0345428 | 0.011373 | 3.04 | 0.0025* |
| targetgender[T.man]*What is your gender?[P.Female] | -0.007712 | 0.011373 | -0.68 | 0.4981 |
| targetmask[T.always]*What is your gender?[P.Female] | -0.006804 | 0.011373 | -0.60 | 0.5500 |
| targetgender[T.man]*targetmask[T.always]*What is your gender?[P.Female] | -0.023747 | 0.011373 | -2.09 | 0.0374* |

#

# Study 2

## Study 2: Changes made to original preregistration prior to data collection

We preregistered this project, piloted, and then made several changes to the preregistration (reported below and reflected in our OSF/preregistration). All changes made to our original preregistration are colored red for transparency within this SOM; the “track changes” appearance is intentional. All alterations to the prereigstration and experiment were made *prior* to collecting data for the Study 2 dataset reported in the manuscript, but after the official preregistration.

We piloted our task on 1/30/2022 -- after preregistering the study -- with 50 participants, and found that data quality was exceptionally low -- of the 52 participants who consented, only 17 met our preregistered exclusion criteria. This told us that we needed a better way to screen for high quality participants. All of our changes to the study/preregistration (after piloting and before collecting our main dataset) were in response to the low data quality. We piloted an additional 50 participants on 2/1/2023 and found that data quality were higher (68% inclusion rate). This led to the changes described below:

1. Only participants who complete a CAPTCHA are eligible to participate. Individuals who do not consent or who do not complete the CAPTCHA will not be included in our participant count.
2. Only participants who identify that they will be evaluating ‘B’ are eligible to participate. Participants who do not answer this question or who answer it incorrectly will not be included in our participant count.
3. Participants who do not respond to the “how often does B wear a mask?” question will be excluded. Participants who do not respond to the “How often do B’s classmates wear a mask” will be excluded.
4. Participants who complete <80% of the study will be excluded.
5. We now force ‘correct’ responses for our three attention checks, and so these are no longer exclusion criteria.

##

## Study 2: How to read this section of the supplement

For the most part, this portion of the SOM is an annotated version of our pre registration, with full reporting of all results. Because of this, there is some redundancy between the main manuscript and SOM; we elected to do this to make it easier for reviewers and readers to connect our research to our preregistration. For readability, we have sometimes changed the order of the preregistration content.

Finally, there are a handful of post-hoc and non-pre registered analyses, clearly labeled as such, that are available in this section of the SOM.

## Study 2 Exclusions and inclusions Dependent Variables, Processing, and Exclusions

### Attention checks (these are exclusion criteria):

Participants who fail or fail to answer any of the following attention checks will be excluded; this is one of our critical manipulations, and therefore no errors are permissible

1. Identifying whether the target always/never wore masks
2. Identifying whether the classmates always/never wore masks.

Participants who fail ~~more than two of the~~ both of the following attention checks will also be excluded; while these are important contextual factors for making judgments about the target, we can tolerate a small amount of error in responding to them:

1. Identifying that the target was a student
2. ~~Selecting our 3 attention check items in each of our scales~~
3. Identifying that the target was 30 years old

###

### Study 2 Final Participant Exclusions

###

| **Sample** | *n* | Total *N* |
| --- | --- | --- |
| Consented, didn’t do pilot, met inclusion criteria (CAPTCHA, B question) | 2389 | 2389 |
| Completed less than 80% of task | 81 | 2308 |
| Didn’t respond to at least one of the two mask memory checks | 0 | 2308 |
| Responded wrong to BOTH the student AND age questions | 86 | 2222 |
| Responded wrong or failed to complete to at least one of the two mask memory checks | 757 | 1465 |
| Final: |  | 1465 |

##

## Study 2 Participants per cell (Classroom fit, must have min of 60)

| Sample size | **None** | **B** | **Classmate** | **Professor** |
| --- | --- | --- | --- | --- |
| **Always/Always** | 83 | 87 | 80 | 89 |
| **Always/Never** | 103 | 103 | 95 | 96 |
| **Never/Always** | 96 | 101 | 103 | 94 |
| **Never/Never** | 80 | 84 | 73 | 98 |

### Target Gender:

Participants will be asked to guess the target’s gender.

## Study 2 Distribution of Genders

- Number of non-binary or non-responses: 150
- Number of women in gender-specific samples = 800
- Number of men in gender-specific samples = 513

## Study 2 Cronbach’s Alpha

###

### Classroom Fit

As in our previous work, we intend to analyze the three questions as a single scale (by taking an average). These items ask (1) how well the target fits in with their classmates; (2) whether the target’s classmates are likely to invite them to the class study group; and (3) how much the professor likes the target.

#### **Alpha**

- .7089

###

### Self Presentation Scale

This scale contains 10 items, based off of Louis, Crum, & Markus (2022)’s Self-Presentation scale.

#### **Alpha**

- .8912

###

### Social Acceptance Scale

This scale contains 7 items, based off of Keyes (1998) Experiment 1

#### **Reverse code**

- 1 (unreliable), 3 (self-centered), 4 (not trustworthy), 5 (for themselves), 6 (dishonest)

#### **Alpha**

- .8136

###

### Social Contribution Scale

This scale contains 6 items, based off of Keyes (1998) Experiment 1

#### **Reverse code**

- 3 (do not produce worthwhile), 4 (no time/energy), 6 (nothing important to contribute)

#### **Alpha**

- .7353

###

## Participant characteristics

Participants will report their gender, their mask-wearing behavior, and their political orientation.


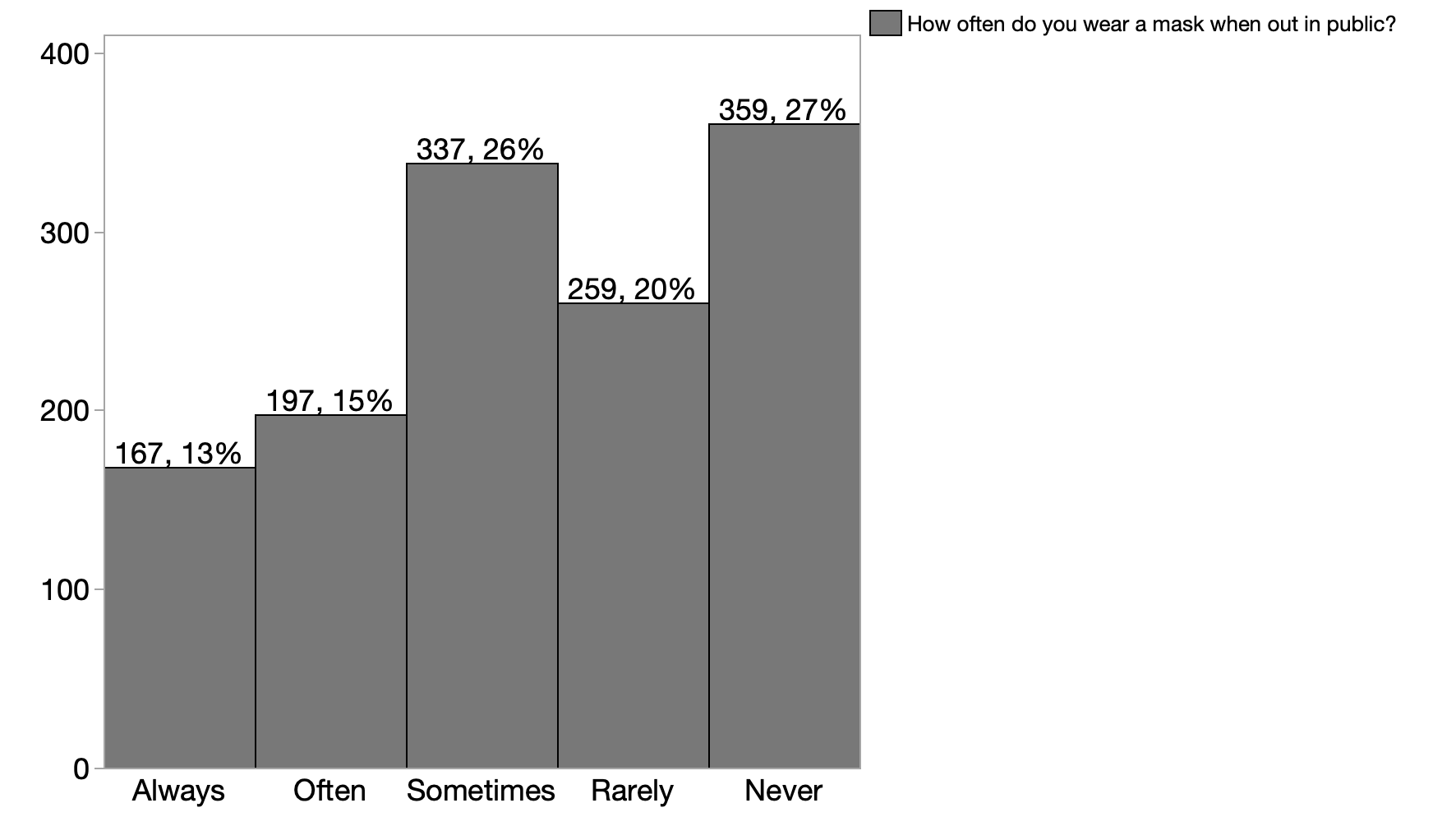


### Figure S2.1. Distribution of participant mask-wearing behavior

labels are absolute number of participants and overall percent of participants


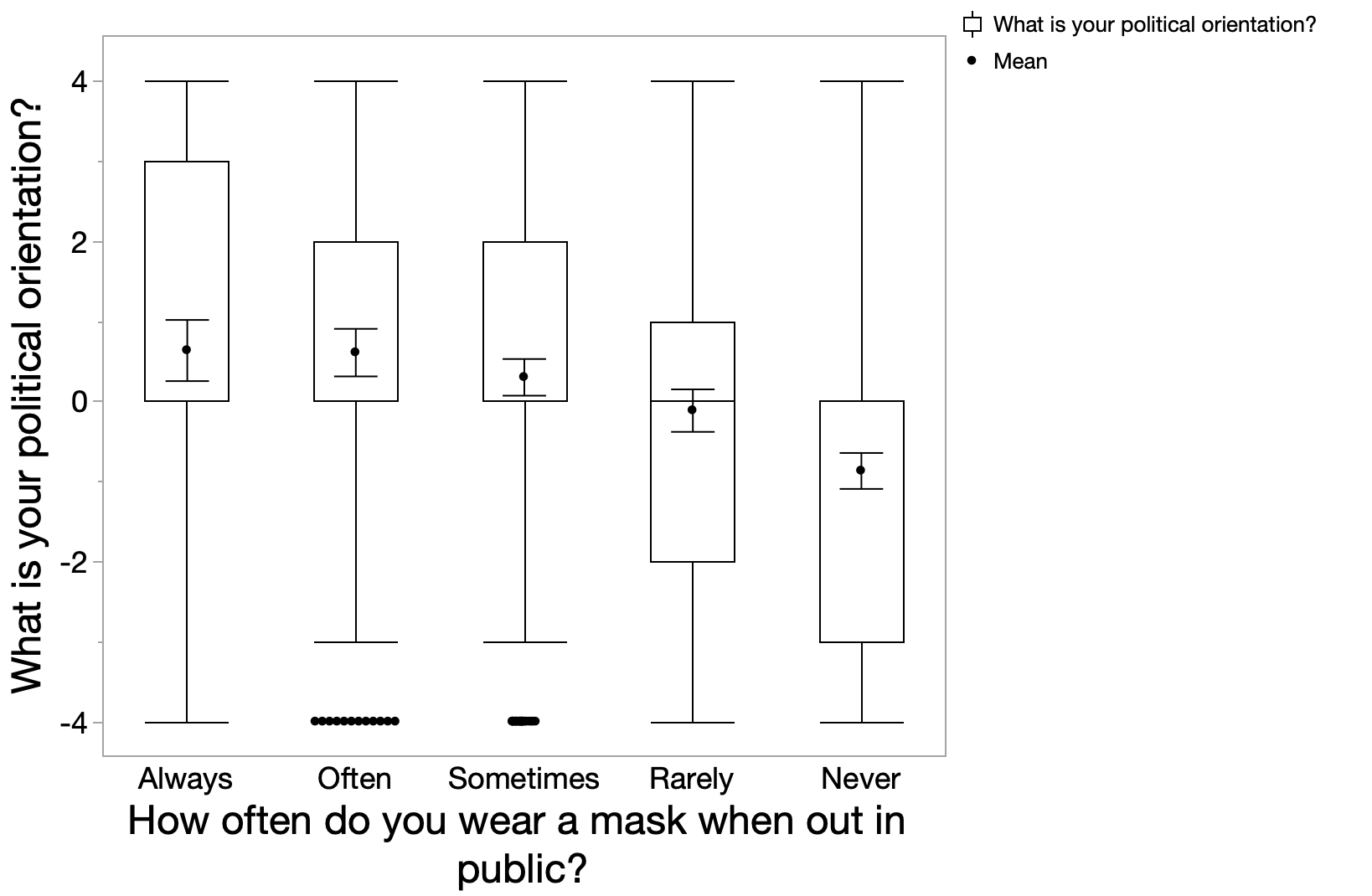


### Figure S2.2. Political orientation and masking.

Political Orientation (positive scores = more liberal; negative scores = more conservative) by participant masking behavior (x-axis). Mean is indicated with a single dot


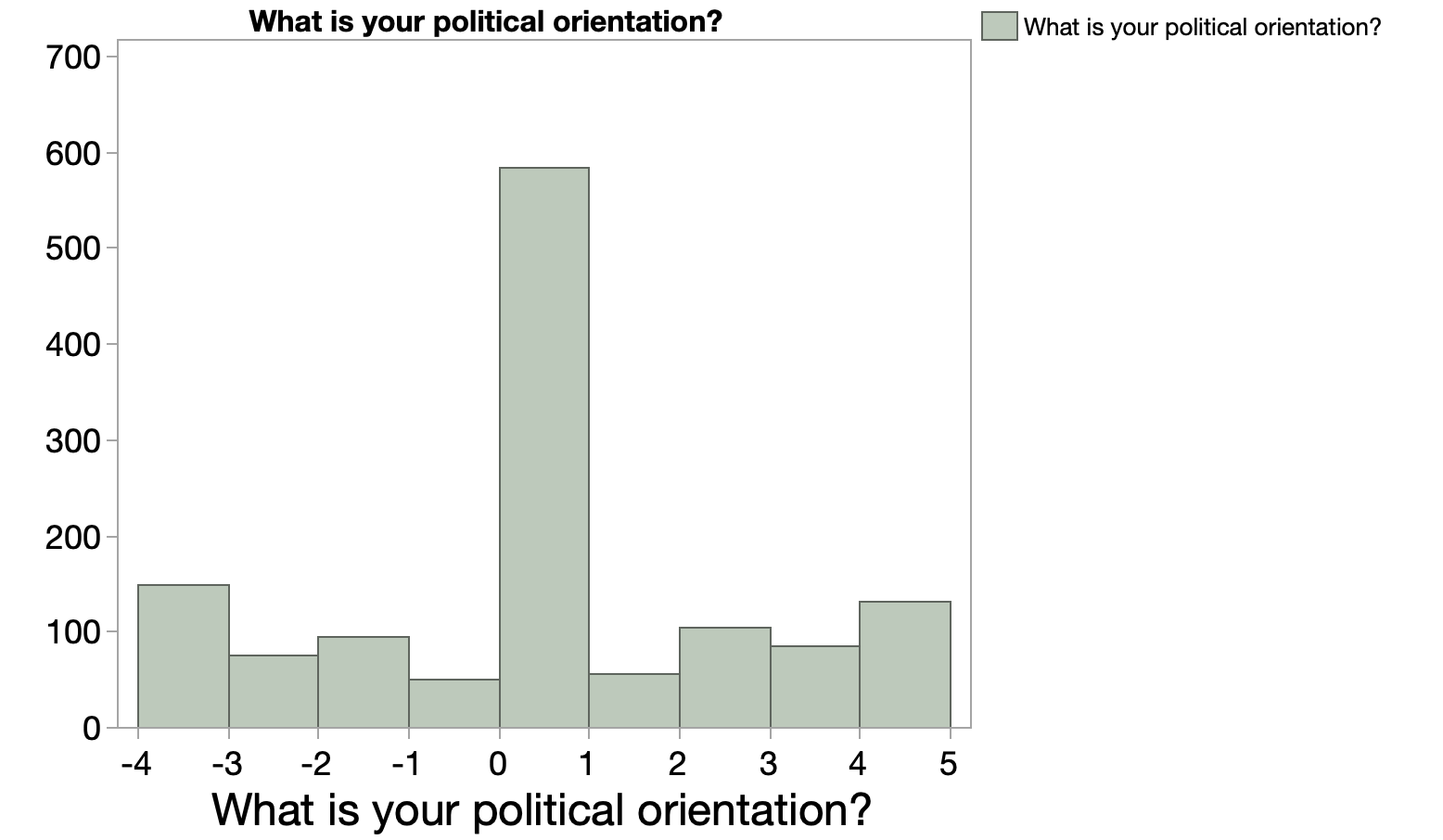


### Figure S2.3. Histogram of political orientation.

## 3) Specific Analyses, by preregistered research question

Note from above: as a reminder, these questions are not listed in order of importance or priority; some of these represent very minor theoretical/applied progress.

## Question 1

####

**A minor extension of our earlier work identifying the effect size of mask-conformity on classroom fit) -- the extension is that in the present study, classroom context is not specified to be an engineering context.**

1. We will first consider the participants who did not learn anything about immunity status. These participants are closest to our previous work. We will then repeat the analyses below for each condition of the immunity-manipulation, in order to develop effect size estimates of the effect of mask conformity in each context.
   1. We will predict perceived Classroom Fit from B’s mask-wearing behavior, the class’s mask-wearing behavior, and their interaction.

##### ClassFit ~ TargetMask*ClassMask (No Immune Status Info Given)

| **Term** | **Estimate** | **Std Error** | **t Ratio** | **Prob>\|t\|** |
| --- | --- | --- | --- | --- |
| Intercept | 0.1562103 | 0.016387 | 9.53 | <.0001* |
| Target Mask[Always] | 0.0804137 | 0.016387 | 4.91 | <.0001* |
| Class Mask[Always] | -0.057456 | 0.016387 | -3.51 | 0.0005* |
| TargetMask[Always]*Class Mask[Always] | 0.2619971 | 0.016387 | 15.99 | <.0001* |

| **Source** | **Nparm** | **DF** | **Sum of Squares** | **F Ratio** | **Prob > F** | η_p_^2^ |
| --- | --- | --- | --- | --- | --- | --- |
| Target Mask | 1 | 1 | 2.315952 | 24.0806 | <.0001* | .06303 |
| Class Mask | 1 | 1 | 1.182350 | 12.2938 | 0.0005* | .03320 |
| Target Mask*Class Mask | 1 | 1 | 24.584531 | 255.6233 | <.0001* | .41658 |

##### ClassFit ~ TargetMask*ClassMask (Full dataset)

| **Term** | **Estimate** | **Std Error** | **t Ratio** | **Prob>\|t\|** |
| --- | --- | --- | --- | --- |
| Intercept | 0.1069031 | 0.008879 | 12.04 | <.0001* |
| Target Mask[Always] | 0.0850088 | 0.008879 | 9.57 | <.0001* |
| Class Mask[Always] | -0.037212 | 0.008879 | -4.19 | <.0001* |
| Target Mask[Always]*Class Mask[Always] | 0.2202069 | 0.008879 | 24.80 | <.0001* |

| **Source** | **Nparm** | **DF** | **Sum of Squares** | **F Ratio** | **Prob > F** |
| --- | --- | --- | --- | --- | --- |
| Target Mask | 1 | 1 | 10.519011 | 91.6541 | <.0001* |
| Class Mask | 1 | 1 | 2.015662 | 17.5628 | <.0001* |
| Target Mask*Class Mask | 1 | 1 | 70.584535 | 615.0165 | <.0001* |

- 1. To better understand any interaction of target- and class-masking behaviors, we will conduct post-hoc Tukey’s HSD in order to compare perceptions of masking for each of the four levels (never-never, always-always, always-never, never-always)

##### Tukey’s HSD No Immune Info Given

| **Level** |  |  |  | **Mean** |
| --- | --- | --- | --- | --- |
| Masked Conformer | A |  |  | 0.4411647 |
| Unmasked Conformer | A |  |  | 0.3952500 |
| Masked Deviant |  | B |  | 0.0320833 |
| Unmasked Deviant |  |  | C | -0.2436570 |

| Targ/Class | Targ/Class | Comparison | d | lower | upper | Tukey HSD |
| --- | --- | --- | --- | --- | --- | --- |
| Masked Conformer  Always/Always | Unmasked Deviant Never/Always | Masking constant, effect of conformity | 2.21 | 1.88 | 2.54 | *p*<.0001 |
| Unmasked Conformer  Never/Never | Unmasked Deviant Never/Always | Masking constant, effect of conformity | 2.06 | 1.73 | 2.39 | *p*<.0001 |
| Masked Deviant  Always/Never | Unmasked Deviant Never/Always | Conformity constant, effect of masking | .89 | .60 | 1.17 | *p*<.0001 |
| Masked Conformer  Always/Always | Masked Deviant  Always/Never | Masking constant, effect of conformity | 1.32 | 1.01 | 1.62 | *p*<.0001 |
| Unmasked Conformer  Never/Never | Masked Deviant  Always/Never | Total opposites | 1.17 | .86 | 1.48 | *p*<.0001 |
| Masked Conformer  Always/Always | Unmasked Conformer  Never/Never | Conformity constant, effect of masking | .15 | -.16 | .455 | *p* = .78 |

##### Tukey’s HSD Full Dataset

| **Level** |  |  |  |  | **Mean** |
| --- | --- | --- | --- | --- | --- |
| Masked Conformer | A |  |  |  | 0.3749066 |
| Unmasked Conformer |  | B |  |  | 0.2793134 |
| Masked Deviant |  |  | C |  | 0.0089171 |
| Unmasked Deviant |  |  |  | D | -0.2355248 |

| **Level** | **- Level** | **Difference** | **Std Err Dif** | **Lower CL** | **Upper CL** | **p-Value** |  |
| --- | --- | --- | --- | --- | --- | --- | --- |
| Masked Conformer | Unmasked Deviant | 0.6104314 | 0.0250527 | 0.5459942 | 0.6748685 | <.0001* |  |
| Unmasked Conformer | Unmasked Deviant | 0.5148382 | 0.0251333 | 0.4501939 | 0.5794825 | <.0001* |  |
| Masked Conformer | Masked Deviant | 0.3659895 | 0.0250966 | 0.3014394 | 0.4305396 | <.0001* |  |
| Unmasked Conformer | Masked Deviant | 0.2703963 | 0.0251770 | 0.2056395 | 0.3351532 | <.0001* |  |
| Masked Deviant | Unmasked Deviant | 0.2444419 | 0.0240911 | 0.1824781 | 0.3064056 | <.0001* |  |
| Masked Conformer | Unmasked Conformer | 0.0955932 | 0.0260987 | 0.0284657 | 0.1627206 | 0.0015* |  |

##### Figures

### Figure S2.4. Bat chart of classroom fit by masking behavior and immune-status for Study 2.

***
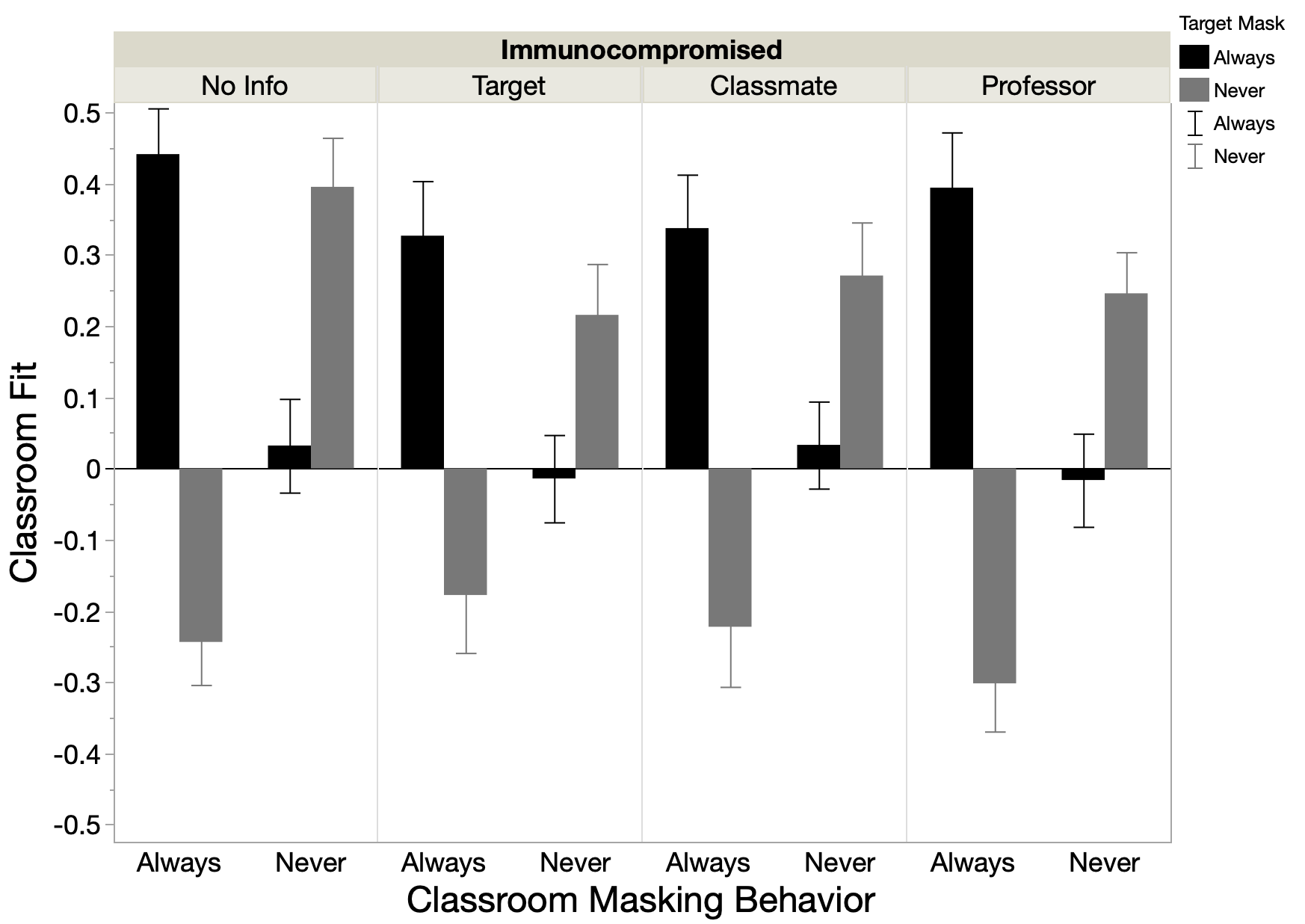
***

## Question 2

####

**A replication of our earlier work identifying that mask-wearing is gendered. The replication component would be to demonstrate (a) that participants who identify as women are more likely to wear masks and (b) that targets who wear masks are more likely to be assumed to be women. Our previous work also found an unexpected effect of classroom masking behavior on the perceived gender of our target -- we would measure whether this still occurs.**

1. For the purpose of replicating our original finding that women are more likely to wear masks, we will predict Participant.Mask.Wearing ~ Participant.Gender
   1. No significant effect

##### Participant.Mask.Wearing ~ Participant.Gender (Full Dataset)

| **Term** | **Estimate** | **Std Error** | **t Ratio** | **Prob>\|t\|** |
| --- | --- | --- | --- | --- |
| Intercept | -0.340417 | 0.038196 | -8.91 | <.0001* |
| Binary Participant Gender[Female] | -0.007083 | 0.038196 | -0.19 | 0.8529 |

1. For the purposes of replicating the finding that masking behavior is perceived to be gendered, we will first focus our analyses on the condition where immunity status is unknown.
   1. Does mask wearing behavior impact the perceived gender of the individual? To test this, we will predict Perceived.Target.Gender ~ Target.Mask.

##### Perceived.Target.Gender ~ Target.Mask

###### Full Dataset

| **Term** | **Estimate** | **Std Error** | **t Ratio** | **Prob>\|t\|** |
| --- | --- | --- | --- | --- |
| Intercept | 0.3953463 | 0.014486 | 27.29 | <.0001* |
| Target Mask[Always] | -0.041685 | 0.014486 | -2.88 | 0.0041* |

*d* = .15 [.05, .25]

###### Immune Status Unknown

**Parameter Estimates**

| **Term** | **Estimate** | **Std Error** | **t Ratio** | **Prob>\|t\|** |
| --- | --- | --- | --- | --- |
| Intercept | 0.3797848 | 0.028914 | 13.13 | <.0001* |
| Target Mask[Always] | -0.054478 | 0.028914 | -1.88 | 0.0604 |

*d* = .198 [-.008, .40]

###### Target is immunocompromised

**Parameter Estimates**

| **Term** | **Estimate** | **Std Error** | **t Ratio** | **Prob>\|t\|** |
| --- | --- | --- | --- | --- |
| Intercept | 0.4197103 | 0.028382 | 14.79 | <.0001* |
| Target Mask[Always] | -0.005827 | 0.028382 | -0.21 | 0.8374 |

*d* = .02 [-.18, .22]

###### Classmate is immunocompromised

**Parameter Estimates**

| **Term** | **Estimate** | **Std Error** | **t Ratio** | **Prob>\|t\|** |
| --- | --- | --- | --- | --- |
| Intercept | 0.3682234 | 0.030118 | 12.23 | <.0001* |
| Target Mask[Always] | -0.082729 | 0.030118 | -2.75 | 0.0063* |

*d* = .29 [.083,.50]

###### Professor is immunocompromised

| **Term** | **Estimate** | **Std Error** | **t Ratio** | **Prob>\|t\|** |
| --- | --- | --- | --- | --- |
| Intercept | 0.4129653 | 0.028569 | 14.45 | <.0001* |
| Target Mask[Always] | -0.025643 | 0.028569 | -0.90 | 0.3700 |

*d* = .092 [-.11,.29]

- 1. Do target and class masking behavior interact in predicting the perceived gender of the individual? To test this, we will construct the following model
  2. We will conduct follow-up Tukey HSD tests as needed to identify the nature of the interaction.

#### **Perceived.Target.gender ~ Target.Mask * Class.Mask**

##### Perceived.Target.gender ~ Target.Mask * Class.Mask (Full Dataset)

**Parameter Estimates**

| **Term** | **Estimate** | **Std Error** | **t Ratio** | **Prob>\|t\|** |
| --- | --- | --- | --- | --- |
| Intercept | 0.3961325 | 0.014486 | 27.35 | <.0001* |
| Target Mask[Always] | -0.037816 | 0.014486 | -2.61 | 0.0091* |
| Class Mask[Always] | 0.0483147 | 0.014486 | 3.34 | 0.0009* |
| Target Mask[Always]*Class Mask[Always] | 0.0125401 | 0.014486 | 0.87 | 0.3868 |

| **Source** | **Nparm** | **DF** | **Sum of Squares** | **F Ratio** | **Prob > F** |
| --- | --- | --- | --- | --- | --- |
| Target Mask | 1 | 1 | 2.0782412 | 6.8150 | 0.0091* |
| Class Mask | 1 | 1 | 3.3924185 | 11.1244 | 0.0009* |
| Target Mask*Class Mask | 1 | 1 | 0.2285347 | 0.7494 | 0.3868 |

##### Perceived.Target.gender and masking: Tukey HSD (Full Dataset)

| **Level** |  |  | **Mean** |
| --- | --- | --- | --- |
| Unmasked Deviant | A |  | 0.46972292 |
| Masked Conformer | A |  | 0.41917160 |
| Unmasked Conformer | A | B | 0.39817365 |
| Masked Deviant |  | B | 0.29746193 |

| **Level** | **- Level** | **Difference** | **Std Err Dif** | **Lower CL** | **Upper CL** | **p-Value** |  |
| --- | --- | --- | --- | --- | --- | --- | --- |
| Unmasked Deviant | Masked Deviant | 0.1722610 | 0.0392700 | 0.071256 | 0.2732659 | <.0001* |  |
| Masked Conformer | Masked Deviant | 0.1217097 | 0.0409416 | 0.016405 | 0.2270141 | 0.0159* |  |
| Unmasked Conformer | Masked Deviant | 0.1007117 | 0.0410734 | -0.004932 | 0.2063551 | 0.0681 |  |
| Unmasked Deviant | Unmasked Conformer | 0.0715493 | 0.0410021 | -0.033911 | 0.1770093 | 0.3008 |  |
| Unmasked Deviant | Masked Conformer | 0.0505513 | 0.0408701 | -0.054569 | 0.1556719 | 0.6034 |  |
| Masked Conformer | Unmasked Conformer | 0.0209979 | 0.0426058 | -0.088587 | 0.1305829 | 0.9607 |  |

| Targ/Class | Targ/Class | Comparison | d | lower | upper | Tukey HSD |
| --- | --- | --- | --- | --- | --- | --- |
| Masked Conformer  Always/Always | Unmasked Deviant Never/Always | Masking constant, effect of conformity | .092 | -.054 | .23662 | *p* = .60 |
| Unmasked Conformer  Never/Never | Unmasked Deviant Never/Always | Masking constant, effect of conformity | .13 | -.016 | .275 | *p* = .30 |
| Masked Deviant  Always/Never | Unmasked Deviant Never/Always | Conformity constant, effect of masking | .312 | .17 | .07 | *p*<.0001 |
| Masked Conformer  Always/Always | Masked Deviant  Always/Never | Masking constant, effect of conformity | .22 | .075 | .365 | *p* = .0159 |
| Unmasked Conformer  Never/Never | Masked Deviant  Always/Never | Total opposites | .182 | .04 | .33 | *p* = .0681 |
| Masked Conformer  Always/Always | Unmasked Conformer  Never/Never | Conformity constant, effect of masking | .038 | -.11 | .189 | *p* = .96 |

##### Perceived.Target.gender and masking: Figure (Full Dataset)


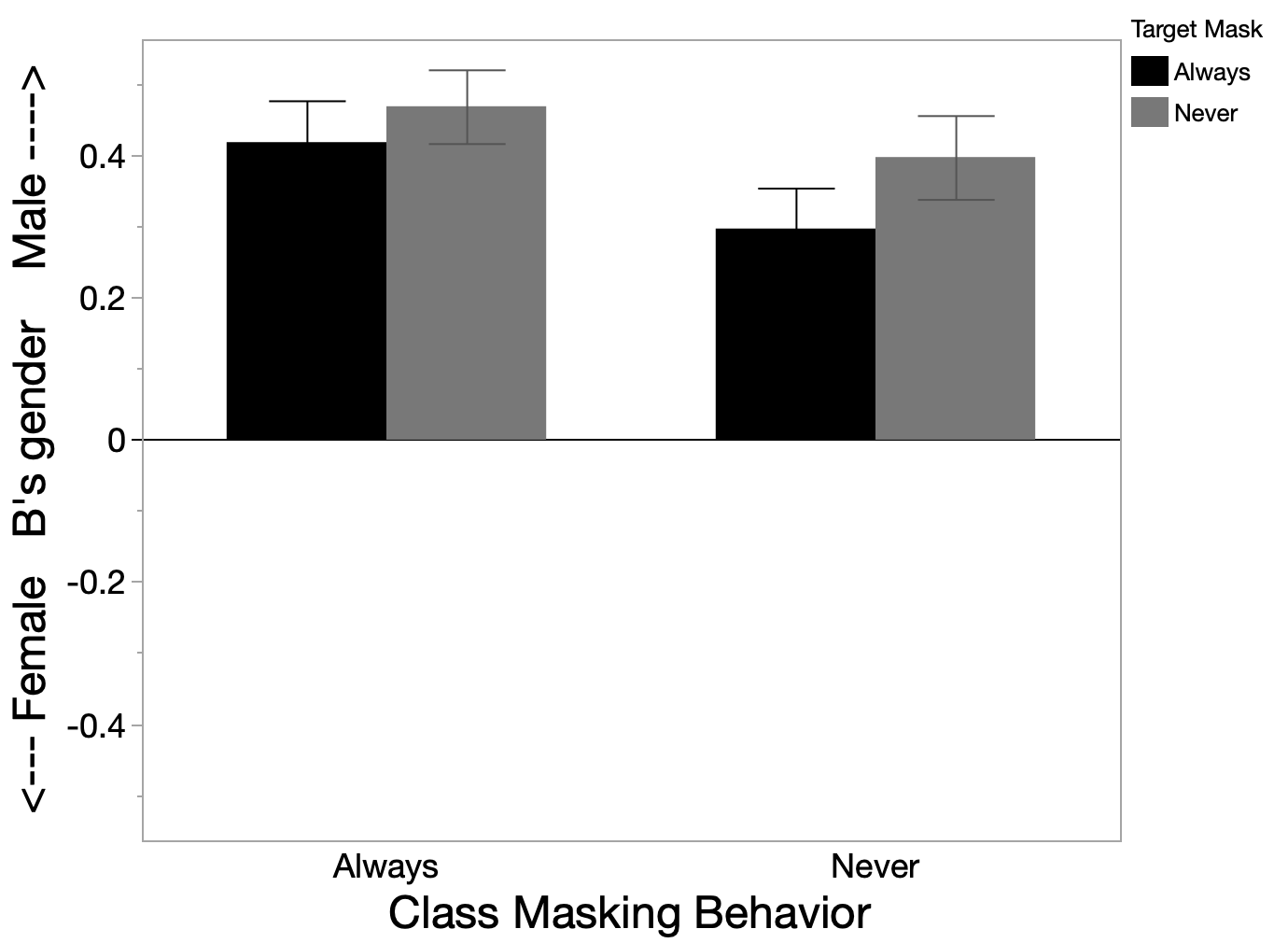


### Figure S2.5. Bar chart of perceived gender by masking behavior for Study 2.

##### Perceived.Target.gender ~ Target.Mask * Class.Mask (Immune Status Unknown)

**Parameter Estimates**

| **Term** | **Estimate** | **Std Error** | **t Ratio** | **Prob>\|t\|** |
| --- | --- | --- | --- | --- |
| Intercept | 0.380851 | 0.028891 | 13.18 | <.0001* |
| Target Mask[Always] | -0.048553 | 0.028891 | -1.68 | 0.0937 |
| Class Mask[Always] | 0.0674552 | 0.028891 | 2.33 | 0.0201* |
| Target Mask[Always]*Class Mask[Always] | 0.0288011 | 0.028891 | 1.00 | 0.3195 |

**Effect Tests**

| **Source** | **Nparm** | **DF** | **Sum of Squares** | **F Ratio** | **Prob > F** |
| --- | --- | --- | --- | --- | --- |
| Target Mask | 1 | 1 | 0.8443118 | 2.8243 | 0.0937 |
| Class Mask | 1 | 1 | 1.6296710 | 5.4515 | 0.0201* |
| Target Mask*Class Mask | 1 | 1 | 0.2970889 | 0.9938 | 0.3195 |

##### Perceived.Target.gender and masking: Figure (No Immune Info Known)


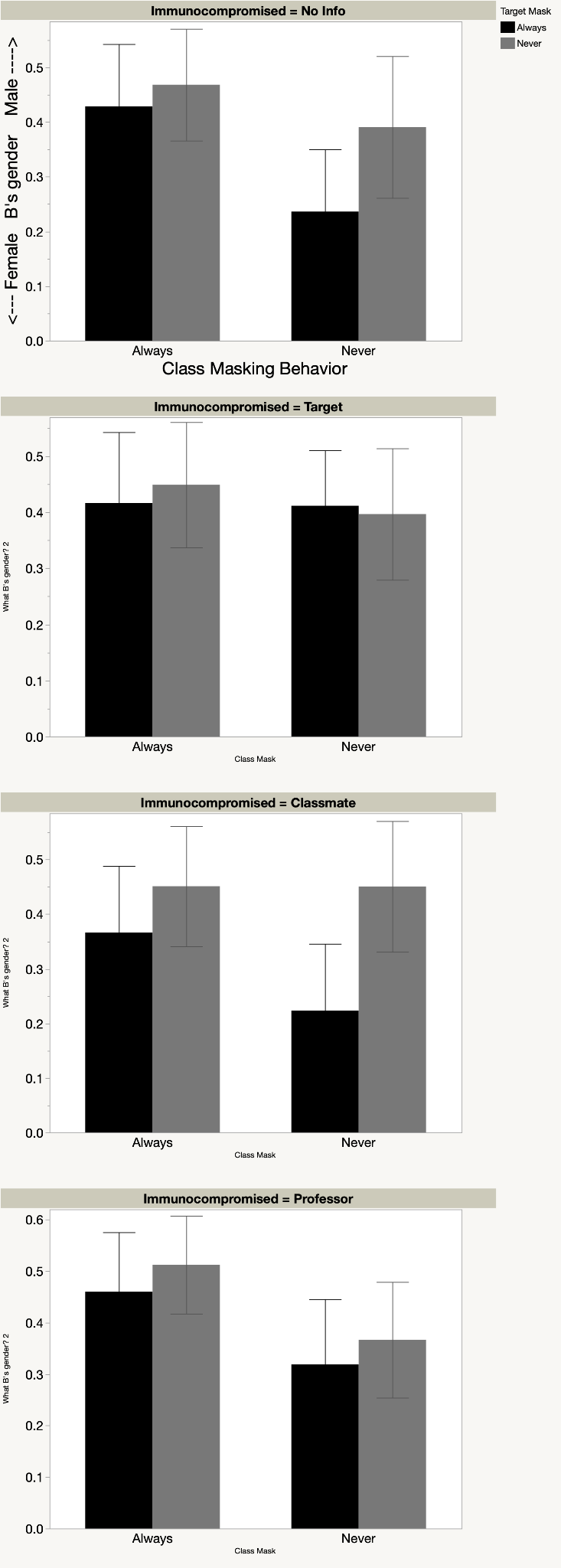


### Figure S2.6. Bar chart of perceived gender by masking behavior for only individuals who received no immune-status info for Study 2.

## Question 3

**An extension of our earlier work to ask whether immune-status (and/or its interaction with masking behavior) is gendered. Specifically, we would ask whether particular immune statuses are gendered.**

1. To test this, we will first ask whether Perceived.Target.Gender is predicted by immune-status. We will conduct an omnibus analysis as follows:

##### Perceived.Target.Gender ~ Immune.Status.Condition

**Effect Tests**

| **Source** | **Nparm** | **DF** | **Sum of Squares** | **F Ratio** | **Prob > F** |
| --- | --- | --- | --- | --- | --- |
| Immunocompromised | 3 | 3 | 0.75377136 | 0.8140 | 0.4861 |

- 1. If there is reason to believe there is any effect whatsoever of I-S Condition on perceived target gender (e.g., there are visible mean differences; the omnibus analysis above is significant), we will next aim to explore those differences.
     1. Assuming our perceived target gender data meet the criteria for continuous analysis, we would like to approach this question using Dunnet’s Mean Comparison, which allows for a comparison to a control while correcting for multiple comparisons. There are two possible controls, motivated below:
        1. One possibility is to treat the ‘no immunity information’ as a control condition. This would allow us to ask about the impact of learning about immunity information, relative to receiving no information about it.

**No impact**

| **Level** | **Abs(Dif)-LSD** | **p-Value** |
| --- | --- | --- |
| Target | -0.06 | 0.6524 |
| Professor | -0.06 | 0.7515 |
| No Info | -0.1 | 1.0000 |
| Classmate | -0.08 | 0.9666 |

- - - 1. A second possibility is to treat “B is immunocompromised” as the ‘control’. This condition would only be a control in the statistical sense (i.e. not theoretically). If being immunocompromised causes changes in perceived vulnerability, we would make the prediction that B’s perceived gender would only be impacted when B is immunocompromised, not when other individuals in the class are immunocompromised.

##### Perceived.Target.Gender ~ Target.Mask * Class.Mask * Immunity.Status

1. We will next ask whether perceived target gender is impacted by immune-status, target masking behavior, and classroom masking behavior (Perceived.Target.Gender ~ Target.Mask * Class.Mask * Immunity.Status). Given the complexity of 3-way interactions, we will visualize any interactions that emerge, and will conduct post-hoc mean comparisons to explore those interactions.

| **Source** | **Nparm** | **DF** | **Sum of Squares** | **F Ratio** | **Prob > F** |
| --- | --- | --- | --- | --- | --- |
| Immunocompromised | 3 | 3 | 0.5749322 | 0.6278 | 0.5971 |
| Target Mask | 1 | 1 | 2.1991066 | 7.2040 | 0.0074* |
| Immunocompromised*Target Mask | 3 | 3 | 1.0727291 | 1.1714 | 0.3193 |
| Class Mask | 1 | 1 | 3.2429089 | 10.6234 | 0.0011* |
| Immunocompromised*Class Mask | 3 | 3 | 0.8165175 | 0.8916 | 0.4448 |
| Target Mask*Class Mask | 1 | 1 | 0.2392462 | 0.7837 | 0.3761 |
| Immunocompromised*Target Mask*Class Mask | 3 | 3 | 0.5684715 | 0.6207 | 0.6016 |

## Question 4

####

An extension of our earlier work to enrich our notion of ‘fit’ as an outcome variable. Thus, in addition to treating ‘classroom fit’ as a key DV, we will also add three new DVs assessing different elements of fit and self-presentation from the health psychology literature.

1. To test this question, we will conduct the analyses described in Question 1, but will predict each of our three new DVs (instead of Classroom Fit).

##### SelfPresentation ~ TargetMask*ClassMask (Full Dataset)

| **Term** | **Estimate** | **Std Error** | **t Ratio** | **Prob>\|t\|** |
| --- | --- | --- | --- | --- |
| Intercept | 0.3768519 | 0.018942 | 19.89 | <.0001* |
| Target Mask[Always] | 0.2504025 | 0.018942 | 13.22 | <.0001* |
| Class Mask[Always] | -0.084278 | 0.018942 | -4.45 | <.0001* |
| Target Mask[Always]*Class Mask[Always] | 0.17787 | 0.018942 | 9.39 | <.0001* |

| **Source** | **Nparm** | **DF** | **Sum of Squares** | **F Ratio** | **Prob > F** | **Partial Eta Sq** |
| --- | --- | --- | --- | --- | --- | --- |
| Target Mask | 1 | 1 | 84.782362 | 174.7522 | <.0001* | .105 |
| Class Mask | 1 | 1 | 9.603992 | 19.7956 | <.0001* | 0.01436 |
| Target Mask*Class Mask | 1 | 1 | 42.779311 | 88.1761 | <.0001* | 0.06093 |

##### SelfPresentation TukeyHSD (Full Dataset)

| **Level** |  |  |  |  | **Mean** |
| --- | --- | --- | --- | --- | --- |
| Masked Conformer | A |  |  |  | 0.7208469 |
| Masked Deviant |  | B |  |  | 0.5336619 |
| Unmasked Conformer |  |  | C |  | 0.3885970 |
| Unmasked Deviant |  |  |  | D | -0.1356982 |

##### Self Presentation Effect Sizes (Full Dataset)

| Targ/Class | Targ/Class | Comparison | d | lower | upper | Tukey HSD |
| --- | --- | --- | --- | --- | --- | --- |
| Masked Conformer  Always/Always | Unmasked Deviant Never/Always | Masking constant, effect of conformity | 1.23 | 1.07 | 1.39 | *p*<.0001 |
| Unmasked Conformer  Never/Never | Unmasked Deviant Never/Always | Masking constant, effect of conformity | .75 | .59956 | .90561 | *p*<.0001 |
| Masked Deviant  Always/Never | Unmasked Deviant Never/Always | Conformity constant, effect of masking | .96 | .812 | 1.11 | *p*<.0001 |
| Masked Conformer  Always/Always | Masked Deviant  Always/Never | Masking constant, effect of conformity | .27 | .11722 | .42015 | *p* = .0029 |
| Unmasked Conformer  Never/Never | Masked Deviant  Always/Never | Total opposites | .21 | .057 | .35863 | *p* = .0336 |
| Masked Conformer  Always/Always | Unmasked Conformer  Never/Never | Conformity constant, effect of masking | .48 | .32 | .63525 | *p*<.0001 |

##### SocialContributuion ~ TargetMask*ClassMask (Full Dataset)

| **Term** | **Estimate** | **Std Error** | **t Ratio** | **Prob>\|t\|** |
| --- | --- | --- | --- | --- |
| Intercept | 0.7367854 | 0.024266 | 30.36 | <.0001* |
| Target Mask[Always] | 0.3560972 | 0.024266 | 14.68 | <.0001* |
| Class Mask[Always] | -0.099672 | 0.024266 | -4.11 | <.0001* |
| Target Mask[Always]*Class Mask[Always] | 0.0605242 | 0.024266 | 2.49 | 0.0127* |

| **Source** | **Nparm** | **DF** | **Sum of Squares** | **F Ratio** | **Prob > F** | **Partial Eta Square** |
| --- | --- | --- | --- | --- | --- | --- |
| Target Mask | 1 | 1 | 168.95556 | 215.3557 | <.0001* | 0.13837 |
| Class Mask | 1 | 1 | 13.23680 | 16.8720 | <.0001* | 0.01243 |
| Target Mask*Class Mask | 1 | 1 | 4.88083 | 6.2213 | 0.0127* | 0.00462 |

##### Social Contribution Tukey’s (Full Dataset)

Oh hey, masking matters here more than conformity!

| **Level** |  |  |  | **Mean** |
| --- | --- | --- | --- | --- |
| Masked Deviant | A |  |  | 1.1320305 |
| Masked Conformer | A |  |  | 1.0537347 |
| Unmasked Conformer |  | B |  | 0.5408846 |
| Unmasked Deviant |  |  | C | 0.2204918 |

##### Social Contribution effect Sizes (Full Dataset)

| **Level** | **- Level** | **Difference** | **Std Err Dif** | **Lower CL** | **Upper CL** | **p-Value** |
| --- | --- | --- | --- | --- | --- | --- |
| Masked Deviant | Unmasked Deviant | 0.9115387 | 0.0652551 | 0.743682 | 1.079396 | <.0001* |
| Masked Conformer | Unmasked Deviant | 0.8332429 | 0.0690467 | 0.655633 | 1.010853 | <.0001* |
| Masked Deviant | Unmasked Conformer | 0.5911460 | 0.0682176 | 0.415668 | 0.766624 | <.0001* |
| Masked Conformer | Unmasked Conformer | 0.5128501 | 0.0718530 | 0.328021 | 0.697679 | <.0001* |
| Unmasked Conformer | Unmasked Deviant | 0.3203928 | 0.0684290 | 0.144371 | 0.496414 | <.0001* |
| Masked Deviant | Masked Conformer | 0.0782959 | 0.0688372 | -0.098776 | 0.255367 | 0.6664 |

#####

| Targ/Class | Targ/Class | Comparison | d | lower | upper | Tukey HSD |
| --- | --- | --- | --- | --- | --- | --- |
| Masked Conformer  Always/Always | Unmasked Deviant Never/Always | Masking constant, effect of conformity | .94 | .78 | 1.097 | *p*<.0001 |
| Unmasked Conformer  Never/Never | Unmasked Deviant Never/Always | Masking constant, effect of conformity | .36 | .2096 | .51369 | *p*<.0001 |
| Masked Deviant  Always/Never | Unmasked Deviant Never/Always | Conformity constant, effect of masking | 1.029 | .879 | 1.18 | *p*<.0001 |
| Masked Conformer  Always/Always | Masked Deviant  Always/Never | Masking constant, effect of conformity | .0884 | -.06398 | .24074 | *p* = .6664 |
| Unmasked Conformer  Never/Never | Masked Deviant  Always/Never | Total opposites | .667 | .514 | .82 | *p*<.0001 |
| Masked Conformer  Always/Always | Unmasked Conformer  Never/Never | Conformity constant, effect of masking | .579 | .418 | .739 | *p*<.0001 |

##### SocialAcceptance ~ TargetMask*ClassMask (Full Dataset)

| **Term** | **Estimate** | **Std Error** | **t Ratio** | **Prob>\|t\|** |
| --- | --- | --- | --- | --- |
| Intercept | 0.0119886 | 0.02631 | 0.46 | 0.6487 |
| Target Mask[Always] | 0.1278101 | 0.02631 | 4.86 | <.0001* |
| Class Mask[Always] | 0.1201065 | 0.02631 | 4.57 | <.0001* |
| Target Mask[Always]*Class Mask[Always] | 0.2410633 | 0.02631 | 9.16 | <.0001* |

| **Source** | **Nparm** | **DF** | **Sum of Squares** | **F Ratio** | **Prob > F** | **Partial Eta Sq** |
| --- | --- | --- | --- | --- | --- | --- |
| Target Mask | 1 | 1 | 21.665785 | 23.5993 | <.0001* | .01547 |
| Class Mask | 1 | 1 | 19.132747 | 20.8402 | <.0001* | .01358 |
| Target Mask*Class Mask | 1 | 1 | 77.073766 | 83.9519 | <.0001* | .05908 |

##### SocialAcceptance Tukey’s (Full Dataset)

| **Level** |  |  |  | **Mean** |
| --- | --- | --- | --- | --- |
| Masked Conformer | A |  |  | 0.5009685 |
| Unmasked Conformer |  | B |  | 0.0051354 |
| Masked Deviant |  |  | C | -0.2213711 |
| Unmasked Deviant |  |  | C | -0.2367783 |

##### SocialAcceptance effect Sizes (Full Dataset)

| **Level** | **- Level** | **Difference** | **Std Err Dif** | **Lower CL** | **Upper CL** | **p-Value** |  |
| --- | --- | --- | --- | --- | --- | --- | --- |
| Masked Conformer | Unmasked Deviant | 0.7377468 | 0.0749243 | 0.545016 | 0.9304771 | <.0001* |  |
| Masked Conformer | Masked Deviant | 0.7223396 | 0.0746553 | 0.530301 | 0.9143780 | <.0001* |  |
| Masked Conformer | Unmasked Conformer | 0.4958331 | 0.0781814 | 0.294725 | 0.6969417 | <.0001* |  |
| Unmasked Conformer | Unmasked Deviant | 0.2419136 | 0.0741740 | 0.051113 | 0.4327139 | 0.0062* |  |
| Unmasked Conformer | Masked Deviant | 0.2265065 | 0.0739023 | 0.036405 | 0.4166077 | 0.0119* |  |
| Masked Deviant | Unmasked Deviant | 0.0154071 | 0.0704476 | -0.165808 | 0.1966219 | 0.9963 |  |

#####

| Targ/Class | Targ/Class | Comparison | d | lower | upper | Tukey HSD |
| --- | --- | --- | --- | --- | --- | --- |
| Masked Conformer  Always/Always | Unmasked Deviant Never/Always | Masking constant, effect of conformity | .76996 | .61381 | .92584 | *p* <.0001 |
| Unmasked Conformer  Never/Never | Unmasked Deviant Never/Always | Masking constant, effect of conformity | .25248 | .10040 | .40446 | *p* = .0062 |
| Masked Deviant  Always/Never | Unmasked Deviant Never/Always | Conformity constant, effect of masking | .01608 | -.12803 | .16018 | *p* = .9963 |
| Masked Conformer  Always/Always | Masked Deviant  Always/Never | Masking constant, effect of conformity | .75388 | .59838 | .90911 | *p* <.0001 |
| Unmasked Conformer  Never/Never | Masked Deviant  Always/Never | Total opposites | .23640 | .08492 | .38779 | *p* = .0119 |
| Masked Conformer  Always/Always | Unmasked Conformer  Never/Never | Conformity constant, effect of masking | .51748 | .35627 | .67851 | *p* <.0001 |

#####

## Question 5

####

We will ask how knowledge of immunity-status interactions with the decision to mask (or not) in shaping perceptions.

1. To test this question, we will conduct the analyses described in Question 3, but with each of our four DVs as the outcome measure (Classroom Fit, Self Presentation, Social Acceptance, Social Contribution). We will conduct appropriate follow-up tests
   1. (DV ~ Target.Mask * Class.Mask * Immunity.Status)

##### ClassroomFit ~ Target.Mask * Class.Mask * Immunity.Status

| **Term** | **Estimate** | **Std Error** | **t Ratio** | **Prob>\|t\|** |
| --- | --- | --- | --- | --- |
| Intercept | 0.1072161 | 0.008848 | 12.12 | <.0001* |
| Immunocompromised[No Info] | 0.0489942 | 0.015385 | 3.18 | 0.0015* |
| Immunocompromised[Target] | -0.019652 | 0.015192 | -1.29 | 0.1960 |
| Immunocompromised[Classmate] | -0.002546 | 0.015586 | -0.16 | 0.8703 |
| Target Mask[Always] | 0.084508 | 0.008848 | 9.55 | <.0001* |
| Immunocompromised[No Info]*Target Mask[Always] | -0.004094 | 0.015385 | -0.27 | 0.7902 |
| Immunocompromised[Target]*Target Mask[Always] | -0.015818 | 0.015192 | -1.04 | 0.2979 |
| Immunocompromised[Classmate]*Target Mask[Always] | -0.004028 | 0.015586 | -0.26 | 0.7961 |
| Class Mask[Always] | -0.038013 | 0.008848 | -4.30 | <.0001* |
| Immunocompromised[No Info]*Class Mask[Always] | -0.019443 | 0.015385 | -1.26 | 0.2065 |
| Immunocompromised[Target]*Class Mask[Always] | 0.0248985 | 0.015192 | 1.64 | 0.1014 |
| Immunocompromised[Classmate]*Class Mask[Always] | -0.009187 | 0.015586 | -0.59 | 0.5557 |
| Target Mask[Always]*Class Mask[Always] | 0.2210902 | 0.008848 | 24.99 | <.0001* |
| Immunocompromised[No Info]*Target Mask[Always]*Class Mask[Always] | 0.0409069 | 0.015385 | 2.66 | 0.0079* |
| Immunocompromised[Target]*Target Mask[Always]*Class Mask[Always] | -0.037563 | 0.015192 | -2.47 | 0.0135* |
| Immunocompromised[Classmate]*Target Mask[Always]*Class Mask[Always] | -0.021749 | 0.015586 | -1.40 | 0.1631 |

| **Source** | **Nparm** | **DF** | **Sum of Squares** | **F Ratio** | **Prob > F** |  |
| --- | --- | --- | --- | --- | --- | --- |
| Immunocompromised | 3 | 3 | 1.275620 | 3.7473 | 0.0107* | .0077 |
| Target Mask | 1 | 1 | 10.351848 | 91.2302 | <.0001* | .05923 |
| Immunocompromised*Target Mask | 3 | 3 | 0.320415 | 0.9413 | 0.4199 | .00195 |
| Class Mask | 1 | 1 | 2.094522 | 18.4589 | <.0001* | .01258 |
| Immunocompromised*Class Mask | 3 | 3 | 0.400152 | 1.1755 | 0.3177 | .00243 |
| Target Mask*Class Mask | 1 | 1 | 70.853482 | 624.4274 | <.0001* | .30116 |
| Immunocompromised*Target Mask*Class Mask | 3 | 3 | 1.414779 | 4.1561 | 0.0061* | .00853 |

- 1. *A priori*, and as noted elsewhere, we suspect that it will be useful to separate our data by immunity status condition (i.e. if immunity status impacts overall perceptions and/or interacts with making above, follow-up analyses would be separated by immunity-status condition). Thus, while we plan to conduct the omnibus analysis described above, the complexity of a 4*2*2 model is not lost on us. We will reduce the dimensionality by taking one (or both) of the following steps for follow-up analyses:
     1. Analyzing each immunity-status condition separately. If there is a three-way interaction of the predictors above, or if it’s clear from descriptives that comparisons across immunity status conditions are not apples-to-apples, this would be the preferred approach.
     2. Collapsing our analysis of masking into ‘conformers’ and ‘deviants’. In our previous work, we found the largest effect of conformity, and it is one of the more central theoretical constructors in our current work -- thus, if justified by the data, we may wish to define ‘conformers’ as those whose masking behavior matches that of their classmates, and ‘deviants’ as those whose masking behavior does not match that of their classmates.

##### Only Tukey-HSD that was sig: Unmasked Conformers (Classroom Fit)

- When the target wore a mask, the only predictor of classroom fit was whether they conformed or deviated from the classroom norm. Immune-statue also didn’t impact perceptions of unmasked deviants, who were rated as having low classroom fit no matter the immune context. However, when either the target or their professor was immunocompromised, unmasked individuals -- even if they were conforming -- were penalized.

**Connecting Letters Report**

This is the detailed report from the Tukey’s HSD

| **Level** |  |  | **Mean** |
| --- | --- | --- | --- |
| No Info | A |  | 0.39525000 |
| Classmate | A | B | 0.27073059 |
| Professor |  | B | 0.24574830 |
| Target |  | B | 0.21551587 |

Levels not connected by same letter are significantly different.

**Ordered Differences Report**

| **Level** | **- Level** | **Difference** | **Std Err Dif** | **Lower CL** | **Upper CL** | **p-Value** |  |
| --- | --- | --- | --- | --- | --- | --- | --- |
| No Info | Target | 0.1797341 | 0.0487235 | 0.053922 | 0.3055459 | 0.0015* |  |
| No Info | Professor | 0.1495017 | 0.0469951 | 0.028153 | 0.2708506 | 0.0087* |  |
| No Info | Classmate | 0.1245194 | 0.0504824 | -0.005834 | 0.2548730 | 0.0671 |  |
| Classmate | Target | 0.0552147 | 0.0499056 | -0.073650 | 0.1840790 | 0.6859 |  |
| Professor | Target | 0.0302324 | 0.0463750 | -0.089515 | 0.1499800 | 0.9148 |  |
| Classmate | Professor | 0.0249823 | 0.0482197 | -0.099529 | 0.1494932 | 0.9547 |  |

####

##### Oneway Analysis of Classroom Fit By Immunocompromised MaskClassification=Masked Conformer

**Means Comparisons**

**Comparisons for all pairs using Tukey-Kramer HSD**

**Connecting Letters Report**

| **Level** |  | **Mean** |
| --- | --- | --- |
| No Info | A | 0.44116466 |
| Professor | A | 0.39408240 |
| Classmate | A | 0.33729167 |
| Target | A | 0.32666667 |

Levels not connected by same letter are significantly different.

**Ordered Differences Report**

| **Level** | **- Level** | **Difference** | **Std Err Dif** | **Lower CL** | **Upper CL** | **p-Value** |  |
| --- | --- | --- | --- | --- | --- | --- | --- |
| No Info | Target | 0.1144980 | 0.0526554 | -0.021458 | 0.2504543 | 0.1325 |  |
| No Info | Classmate | 0.1038730 | 0.0537683 | -0.034957 | 0.2427030 | 0.2167 |  |
| Professor | Target | 0.0674157 | 0.0517391 | -0.066175 | 0.2010061 | 0.5616 |  |
| Professor | Classmate | 0.0567907 | 0.0528713 | -0.079723 | 0.1933047 | 0.7056 |  |
| No Info | Professor | 0.0470823 | 0.0523657 | -0.088126 | 0.1822907 | 0.8053 |  |
| Classmate | Target | 0.0106250 | 0.0531582 | -0.126630 | 0.1478797 | 0.9972 |  |

#####

##### Oneway Analysis of Classroom Fit By Immunocompromised MaskClassification=Masked Deviant

**Means Comparisons**

**Comparisons for all pairs using Tukey-Kramer HSD**

**Connecting Letters Report**

| **Level** |  | **Mean** |
| --- | --- | --- |
| Classmate | A | 0.0330097 |
| No Info | A | 0.0320833 |
| Target | A | -0.0141584 |
| Professor | A | -0.0163475 |

Levels not connected by same letter are significantly different.

**Ordered Differences Report**

| **Level** | **- Level** | **Difference** | **Std Err Dif** | **Lower CL** | **Upper CL** | **p-Value** |  |
| --- | --- | --- | --- | --- | --- | --- | --- |
| Classmate | Professor | 0.0493572 | 0.0451692 | -0.067187 | 0.1659017 | 0.6942 |  |
| No Info | Professor | 0.0484309 | 0.0459483 | -0.070124 | 0.1669854 | 0.7176 |  |
| Classmate | Target | 0.0471681 | 0.0443433 | -0.067245 | 0.1615815 | 0.7118 |  |
| No Info | Target | 0.0462417 | 0.0451366 | -0.070219 | 0.1627021 | 0.7352 |  |
| Target | Professor | 0.0021891 | 0.0453821 | -0.114905 | 0.1192829 | 1.0000 |  |
| Classmate | No Info | 0.0009264 | 0.0449225 | -0.114982 | 0.1168344 | 1.0000 |  |

##### Oneway Analysis of Classroom Fit By Immunocompromised MaskClassification=Unmasked Conformer

**Means Comparisons**

**Comparisons for all pairs using Tukey-Kramer HSD**

**Connecting Letters Report**

| **Level** |  |  | **Mean** |
| --- | --- | --- | --- |
| No Info | A |  | 0.39525000 |
| Classmate | A | B | 0.27073059 |
| Professor |  | B | 0.24574830 |
| Target |  | B | 0.21551587 |

Levels not connected by same letter are significantly different.

**Ordered Differences Report**

| **Level** | **- Level** | **Difference** | **Std Err Dif** | **Lower CL** | **Upper CL** | **p-Value** |  |
| --- | --- | --- | --- | --- | --- | --- | --- |
| No Info | Target | 0.1797341 | 0.0487235 | 0.053922 | 0.3055459 | 0.0015* |  |
| No Info | Professor | 0.1495017 | 0.0469951 | 0.028153 | 0.2708506 | 0.0087* |  |
| No Info | Classmate | 0.1245194 | 0.0504824 | -0.005834 | 0.2548730 | 0.0671 |  |
| Classmate | Target | 0.0552147 | 0.0499056 | -0.073650 | 0.1840790 | 0.6859 |  |
| Professor | Target | 0.0302324 | 0.0463750 | -0.089515 | 0.1499800 | 0.9148 |  |
| Classmate | Professor | 0.0249823 | 0.0482197 | -0.099529 | 0.1494932 | 0.9547 |  |

##### Oneway Analysis of Classroom Fit By Immunocompromised MaskClassification=Unmasked Deviant

**Means Comparisons**

**Comparisons for all pairs using Tukey-Kramer HSD**

**Connecting Letters Report**

| **Level** |  | **Mean** |
| --- | --- | --- |
| Target | A | -0.1777670 |
| Classmate | A | -0.2223509 |
| No Info | A | -0.2436570 |
| Professor | A | -0.3018056 |

Levels not connected by same letter are significantly different.

**Ordered Differences Report**

| **Level** | **- Level** | **Difference** | **Std Err Dif** | **Lower CL** | **Upper CL** | **p-Value** |  |
| --- | --- | --- | --- | --- | --- | --- | --- |
| Target | Professor | 0.1240386 | 0.0524205 | -0.011211 | 0.2592883 | 0.0854 |  |
| Classmate | Professor | 0.0794547 | 0.0534747 | -0.058515 | 0.2174243 | 0.4470 |  |
| Target | No Info | 0.0658900 | 0.0514903 | -0.066960 | 0.1987396 | 0.5764 |  |
| No Info | Professor | 0.0581486 | 0.0524205 | -0.077101 | 0.1933983 | 0.6840 |  |
| Target | Classmate | 0.0445839 | 0.0525631 | -0.091034 | 0.1802016 | 0.8313 |  |
| Classmate | No Info | 0.0213061 | 0.0525631 | -0.114312 | 0.1569238 | 0.9775 |  |

##### SelfPresentation ~ Target.Mask * Class.Mask * Immunity.Status

| **Source** | **LogWorth** |  | **PValue** |
| --- | --- | --- | --- |
| Target Mask | 36.658 |  | 0.00000 |
| Target Mask*Class Mask | 19.658 |  | 0.00000 |
| Class Mask | 4.924 |  | 0.00001 |
| Immunocompromised*Target Mask*Class Mask | 0.568 |  | 0.27025 |
| Immunocompromised | 0.403 |  | 0.39574 |
| Immunocompromised*Class Mask | 0.182 |  | 0.65807 |
| Immunocompromised*Target Mask | 0.177 |  | 0.66473 |

##### Social Contribution ~ Target.Mask * Class.Mask * Immunity.Status

| **Source** | **Nparm** | **DF** | **Sum of Squares** | **F Ratio** | **Prob > F** |
| --- | --- | --- | --- | --- | --- |
| Immunocompromised | 3 | 3 | 3.23127 | 1.3786 | 0.2477 |
| Target Mask | 1 | 1 | 168.40452 | 215.5432 | <.0001* |
| Immunocompromised*Target Mask | 3 | 3 | 1.54299 | 0.6583 | 0.5778 |
| Class Mask | 1 | 1 | 13.70846 | 17.5456 | <.0001* |
| Immunocompromised*Class Mask | 3 | 3 | 7.40443 | 3.1590 | 0.0239* |
| Target Mask*Class Mask | 1 | 1 | 5.03609 | 6.4458 | 0.0112* |
| Immunocompromised*Target Mask*Class Mask | 3 | 3 | 1.84370 | 0.7866 | 0.5014 |

##### Social Acceptance ~ Target.Mask * Class.Mask * Immunity.Status

| **Source** | **Nparm** | **DF** | **Sum of Squares** | **F Ratio** | **Prob > F** |
| --- | --- | --- | --- | --- | --- |
| Immunocompromised | 3 | 3 | 1.519665 | 0.5545 | 0.6452 |
| Target Mask | 1 | 1 | 21.855515 | 23.9226 | <.0001* |
| Immunocompromised*Target Mask | 3 | 3 | 4.881450 | 1.7810 | 0.1489 |
| Class Mask | 1 | 1 | 18.192002 | 19.9126 | <.0001* |
| Immunocompromised*Class Mask | 3 | 3 | 8.239227 | 3.0062 | 0.0294* |
| Target Mask*Class Mask | 1 | 1 | 76.007839 | 83.1968 | <.0001* |
| Immunocompromised*Target Mask*Class Mask | 3 | 3 | 1.231987 | 0.4495 | 0.7177 |

##### Tukey’s HSD self Presentation

**Connecting Letters Report**

| **Level** |  |  |  | **Mean** |
| --- | --- | --- | --- | --- |
| No Info Never | A |  |  | 0.52706254 |
| Classmate Never | A | B |  | 0.49591700 |
| Professor Never | A | B | C | 0.43597299 |
| Target Never | A | B | C | 0.41447028 |
| Classmate Always | A | B | C | 0.29581619 |
| Target Always |  | B | C | 0.27434921 |
| No Info Always |  |  | C | 0.23275805 |
| Professor Always |  |  | C | 0.20948668 |

Levels not connected by same letter are significantly different.

**Ordered Differences Report**

| **Level** | **- Level** | **Difference** | **Std Err Dif** | **Lower CL** | **Upper CL** | **p-Value** |  |
| --- | --- | --- | --- | --- | --- | --- | --- |
| No Info Never | Professor Always | 0.3175759 | 0.0827464 | 0.066387 | 0.5687650 | 0.0033* |  |
| No Info Never | No Info Always | 0.2943045 | 0.0829880 | 0.042382 | 0.5462269 | 0.0096* |  |
| Classmate Never | Professor Always | 0.2864303 | 0.0828724 | 0.034859 | 0.5380020 | 0.0131* |  |
| Classmate Never | No Info Always | 0.2631589 | 0.0831136 | 0.010855 | 0.5154628 | 0.0338* |  |
| No Info Never | Target Always | 0.2527133 | 0.0822778 | 0.002947 | 0.5024801 | 0.0450* |  |
| No Info Never | Classmate Always | 0.2312464 | 0.0838744 | -0.023367 | 0.4858596 | 0.1070 |  |
| Professor Never | Professor Always | 0.2264863 | 0.0811112 | -0.019739 | 0.4727117 | 0.0977 |  |
| Classmate Never | Target Always | 0.2215678 | 0.0824045 | -0.028584 | 0.4717192 | 0.1268 |  |
| Target Never | Professor Always | 0.2049836 | 0.0821357 | -0.044352 | 0.4543188 | 0.1980 |  |
| Professor Never | No Info Always | 0.2032149 | 0.0813577 | -0.043758 | 0.4501884 | 0.1971 |  |
| Classmate Never | Classmate Always | 0.2001008 | 0.0839987 | -0.054890 | 0.4550914 | 0.2509 |  |
| Target Never | No Info Always | 0.1817122 | 0.0823790 | -0.068362 | 0.4317862 | 0.3487 |  |
| Professor Never | Target Always | 0.1616238 | 0.0806332 | -0.083150 | 0.4063979 | 0.4792 |  |
| Professor Never | Classmate Always | 0.1401568 | 0.0822616 | -0.109561 | 0.3898744 | 0.6849 |  |
| Target Never | Target Always | 0.1401211 | 0.0816636 | -0.107781 | 0.3880232 | 0.6769 |  |
| Target Never | Classmate Always | 0.1186541 | 0.0832719 | -0.134130 | 0.3714385 | 0.8458 |  |
| No Info Never | Target Never | 0.1125923 | 0.0826275 | -0.138236 | 0.3634204 | 0.8740 |  |
| No Info Never | Professor Never | 0.0910896 | 0.0816092 | -0.156647 | 0.3388266 | 0.9534 |  |
| Classmate Always | Professor Always | 0.0863295 | 0.0833899 | -0.166813 | 0.3394722 | 0.9690 |  |
| Classmate Never | Target Never | 0.0814467 | 0.0827536 | -0.169764 | 0.3326579 | 0.9767 |  |
| Target Always | Professor Always | 0.0648625 | 0.0817839 | -0.183405 | 0.3131300 | 0.9935 |  |
| Classmate Always | No Info Always | 0.0630581 | 0.0836296 | -0.190812 | 0.3169285 | 0.9952 |  |
| Classmate Never | Professor Never | 0.0599440 | 0.0817370 | -0.188181 | 0.3080688 | 0.9960 |  |
| Target Always | No Info Always | 0.0415912 | 0.0820283 | -0.207418 | 0.2906005 | 0.9996 |  |
| No Info Never | Classmate Never | 0.0311455 | 0.0833598 | -0.221906 | 0.2841969 | 1.0000 |  |
| No Info Always | Professor Always | 0.0232714 | 0.0824983 | -0.227165 | 0.2737074 | 1.0000 |  |
| Professor Never | Target Never | 0.0215027 | 0.0809899 | -0.224354 | 0.2673598 | 1.0000 |  |
| Classmate Always | Target Always | 0.0214670 | 0.0829250 | -0.230264 | 0.2731983 | 1.0000 |  |

##### Tukey’s HSD Social Contribution

| **Level** |  |  |  | **Mean** |
| --- | --- | --- | --- | --- |
| No Info Never | A |  |  | 1.0233533 |
| Classmate Never | A | B |  | 0.8733533 |
| Professor Never | A | B |  | 0.8099251 |
| Target Never | A | B | C | 0.7511905 |
| Target Always |  | B | C | 0.6812261 |
| Professor Always |  | B | C | 0.6432323 |
| No Info Always |  | B | C | 0.5598394 |
| Classmate Always |  |  | C | 0.4885417 |

| **Level** | **- Level** | **Difference** | **Std Err Dif** | **Lower CL** | **Upper CL** | **p-Value** |  |
| --- | --- | --- | --- | --- | --- | --- | --- |
| No Info Never | Classmate Always | 0.5348116 | 0.1054415 | 0.214721 | 0.8549021 | <.0001* |  |
| No Info Never | No Info Always | 0.4635139 | 0.1044638 | 0.146392 | 0.7806363 | 0.0003* |  |
| Classmate Never | Classmate Always | 0.3848116 | 0.1054415 | 0.064721 | 0.7049021 | 0.0066* |  |
| No Info Never | Professor Always | 0.3801210 | 0.1046225 | 0.062517 | 0.6977249 | 0.0070* |  |
| No Info Never | Target Always | 0.3421272 | 0.1032525 | 0.028682 | 0.6555723 | 0.0212* |  |
| Professor Never | Classmate Always | 0.3213834 | 0.1038352 | 0.006170 | 0.6365973 | 0.0420* |  |
| Classmate Never | No Info Always | 0.3135139 | 0.1044638 | -0.003608 | 0.6306363 | 0.0553 |  |
| No Info Never | Target Never | 0.2721628 | 0.1041515 | -0.044011 | 0.5883371 | 0.1520 |  |
| Target Never | Classmate Always | 0.2626488 | 0.1052879 | -0.056975 | 0.5822728 | 0.1985 |  |
| Professor Never | No Info Always | 0.2500857 | 0.1028422 | -0.062114 | 0.5622852 | 0.2268 |  |
| Classmate Never | Professor Always | 0.2301210 | 0.1046225 | -0.087483 | 0.5477249 | 0.3526 |  |
| No Info Never | Professor Never | 0.2134282 | 0.1026827 | -0.098287 | 0.5251437 | 0.4294 |  |
| Target Always | Classmate Always | 0.1926844 | 0.1043986 | -0.124240 | 0.5096088 | 0.5888 |  |
| Classmate Never | Target Always | 0.1921272 | 0.1032525 | -0.121318 | 0.5055723 | 0.5783 |  |
| Target Never | No Info Always | 0.1913511 | 0.1043087 | -0.125300 | 0.5080026 | 0.5965 |  |
| Professor Never | Professor Always | 0.1666928 | 0.1030033 | -0.145996 | 0.4793814 | 0.7393 |  |
| Professor Always | Classmate Always | 0.1546907 | 0.1057538 | -0.166348 | 0.4757289 | 0.8271 |  |
| No Info Never | Classmate Never | 0.1500000 | 0.1043069 | -0.166646 | 0.4666458 | 0.8394 |  |
| Professor Never | Target Always | 0.1286990 | 0.1016115 | -0.179764 | 0.4371625 | 0.9110 |  |
| Classmate Never | Target Never | 0.1221628 | 0.1041515 | -0.194011 | 0.4383371 | 0.9395 |  |
| Target Always | No Info Always | 0.1213867 | 0.1034110 | -0.192540 | 0.4353131 | 0.9393 |  |
| Target Never | Professor Always | 0.1079582 | 0.1044676 | -0.209176 | 0.4250920 | 0.9693 |  |
| Professor Always | No Info Always | 0.0833930 | 0.1047790 | -0.234686 | 0.4014720 | 0.9933 |  |
| No Info Always | Classmate Always | 0.0712977 | 0.1055968 | -0.249264 | 0.3918595 | 0.9976 |  |
| Target Never | Target Always | 0.0699644 | 0.1030955 | -0.243004 | 0.3829331 | 0.9975 |  |
| Classmate Never | Professor Never | 0.0634282 | 0.1026827 | -0.248287 | 0.3751437 | 0.9987 |  |
| Professor Never | Target Never | 0.0587346 | 0.1025249 | -0.252502 | 0.3699711 | 0.9992 |  |
| Target Always | Professor Always | 0.0379937 | 0.1035713 | -0.276419 | 0.3524066 | 1.0000 |  |

##### Tukey’s HSD Social Acceptance

| **Level** |  |  |  | **Mean** |
| --- | --- | --- | --- | --- |
| Target Always | A |  |  | 0.1404901 |
| Classmate Always | A | B |  | 0.1232939 |
| Professor Always | A | B |  | 0.1055130 |
| No Info Never | A | B | C | 0.0543199 |
| No Info Always | A | B | C | -0.0013196 |
| Professor Never | A | B | C | -0.0750605 |
| Target Never |  | B | C | -0.2098739 |
| Classmate Never |  |  | C | -0.2491342 |

| **Level** | **- Level** | **Difference** | **Std Err Dif** | **Lower CL** | **Upper CL** | **p-Value** |  |
| --- | --- | --- | --- | --- | --- | --- | --- |
| Target Always | Classmate Never | 0.3896243 | 0.1083255 | 0.060777 | 0.7184712 | 0.0080* |  |
| Classmate Always | Classmate Never | 0.3724281 | 0.1106719 | 0.036458 | 0.7083979 | 0.0179* |  |
| Professor Always | Classmate Never | 0.3546472 | 0.1087994 | 0.024362 | 0.6849328 | 0.0252* |  |
| Target Always | Target Never | 0.3503641 | 0.1075117 | 0.023988 | 0.6767405 | 0.0253* |  |
| Classmate Always | Target Never | 0.3331679 | 0.1098754 | -0.000384 | 0.6667199 | 0.0505 |  |
| Professor Always | Target Never | 0.3153870 | 0.1079892 | -0.012439 | 0.6432129 | 0.0693 |  |
| No Info Never | Classmate Never | 0.3034541 | 0.1089607 | -0.027321 | 0.6342293 | 0.0996 |  |
| No Info Never | Target Never | 0.2641939 | 0.1081517 | -0.064125 | 0.5925131 | 0.2216 |  |
| No Info Always | Classmate Never | 0.2478146 | 0.1091237 | -0.083455 | 0.5790846 | 0.3105 |  |
| Target Always | Professor Never | 0.2155506 | 0.1064403 | -0.107573 | 0.5386745 | 0.4651 |  |
| No Info Always | Target Never | 0.2085544 | 0.1083159 | -0.120263 | 0.5373721 | 0.5335 |  |
| Classmate Always | Professor Never | 0.1983544 | 0.1088273 | -0.132016 | 0.5287246 | 0.6046 |  |
| Professor Always | Professor Never | 0.1805736 | 0.1069226 | -0.144014 | 0.5051615 | 0.6946 |  |
| Professor Never | Classmate Never | 0.1740737 | 0.1074201 | -0.152025 | 0.5001720 | 0.7379 |  |
| Target Always | No Info Always | 0.1418097 | 0.1081593 | -0.186533 | 0.4701520 | 0.8948 |  |
| Professor Never | Target Never | 0.1348134 | 0.1065994 | -0.188793 | 0.4584203 | 0.9117 |  |
| No Info Never | Professor Never | 0.1293805 | 0.1070867 | -0.195706 | 0.4544666 | 0.9297 |  |
| Classmate Always | No Info Always | 0.1246135 | 0.1105092 | -0.210863 | 0.4600895 | 0.9508 |  |
| Professor Always | No Info Always | 0.1068326 | 0.1086340 | -0.222951 | 0.4366159 | 0.9768 |  |
| Target Always | No Info Never | 0.0861702 | 0.1079949 | -0.241673 | 0.4140133 | 0.9932 |  |
| No Info Always | Professor Never | 0.0737410 | 0.1072525 | -0.251849 | 0.3993305 | 0.9973 |  |
| Classmate Always | No Info Never | 0.0689740 | 0.1103483 | -0.266013 | 0.4039614 | 0.9985 |  |
| No Info Never | No Info Always | 0.0556395 | 0.1087955 | -0.274634 | 0.3859131 | 0.9996 |  |
| Professor Always | No Info Never | 0.0511931 | 0.1084702 | -0.278093 | 0.3804794 | 0.9998 |  |
| Target Never | Classmate Never | 0.0392602 | 0.1084818 | -0.290061 | 0.3685817 | 1.0000 |  |
| Target Always | Professor Always | 0.0349771 | 0.1078321 | -0.292372 | 0.3623262 | 1.0000 |  |
| Classmate Always | Professor Always | 0.0177809 | 0.1101890 | -0.316723 | 0.3522848 | 1.0000 |  |
| Target Always | Classmate Always | 0.0171962 | 0.1097211 | -0.315887 | 0.3502797 | 1.0000 |  |

####

#### **Exploratory Analyses**

1. While not of primary interest, we may analyze some specific items within our scales separately, in addition to analyzing the scale as a whole. For example, our classroom fit scale includes an item assessing how much the professor likes the target -- we might expect that this item would be scored especially high when the target masks and the professor is immunocompromised.

##### Post-hoc correlation of Classroom Fit by Self Presentation & Masking


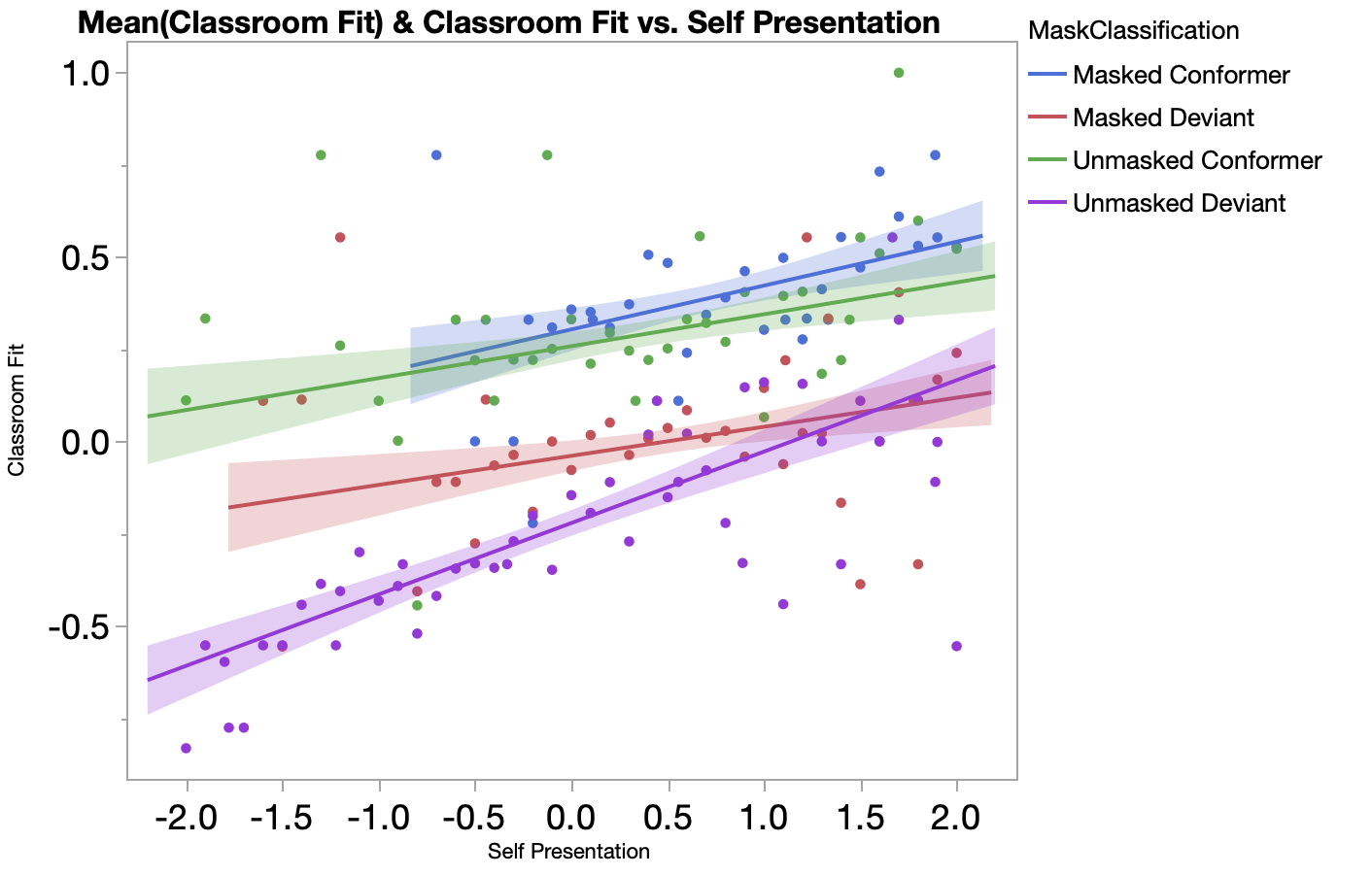


### Figure S2.7. Line chart of Classroom Fit and Self Presentation, by masking status

##

## Secondary DVs

### *Social Acceptance*.

Deviants did not differ by masking behavior: both masked deviants (*M*=-.22) and unmasked deviants (*M*=-.24) were perceived as comparably mistrustful of others (*p*=.996, *d*=.016 [CI*_d_=*-.13, .16]). However, unmasked conformers were perceived as having greater trust than either of the deviants (all *p*<.015, all *d*>.2), and masked conformers were perceived as having the highest level of trust at all (all *p*<.0001, all *d* >.5). These data suggest that conformity is associated with higher levels of perceived trust, and that masked conformers were perceived as feeling the highest levels of trust.

### *Social Contribution*.

Masked deviants (*M*=1.13) and masked conformers (*M*=1.05) had the highest social contribution score and did not differ from one another (*p*=.67, *d*=.09 [CI*_d_=*-.06, .24]), suggesting that masking -- and not conformity -- was the strongest predictors of perceived contribution to society. Unmasked conformers (*M*=.54) had significantly lower scores than either masked deviants (*p* <.0001, *d*=.67 [CI*_d_=*.51,.82]) or masked conformers (*p* <.0001, *d* =.58 [CI*_d_=*.42,.74]). Finally, unmasked deviants had the lowest contribution scores (all *p* <.0001, all *d*>.9)

## Exploratory Analyses: impact of participant masking behavior on the effect of mask conformity

### Classroom Fit

**Parameter Estimates**

| **Term** | **Estimate** | **Std Error** | **t Ratio** | **Prob>\|t\|** |
| --- | --- | --- | --- | --- |
| Intercept | 0.1129171 | 0.009572 | 11.80 | <.0001* |
| Continuous Participant Masking | 0.0079778 | 0.006861 | 1.16 | 0.2451 |
| Target Mask[Always] | 0.087141 | 0.00931 | 9.36 | <.0001* |
| Class Mask[Always] | -0.039001 | 0.009295 | -4.20 | <.0001* |
| Target Mask[Always]*Class Mask[Always] | 0.2334644 | 0.009287 | 25.14 | <.0001* |

### Self Presentation

**Parameter Estimates**

| **Term** | **Estimate** | **Std Error** | **t Ratio** | **Prob>\|t\|** |
| --- | --- | --- | --- | --- |
| Intercept | 0.3908689 | 0.019887 | 19.65 | <.0001* |
| Continuous Participant Masking | 0.0279262 | 0.014254 | 1.96 | 0.0503 |
| Target Mask[Always] | 0.2487539 | 0.019342 | 12.86 | <.0001* |
| Class Mask[Always] | -0.08536 | 0.019312 | -4.42 | <.0001* |
| Target Mask[Always]*Class Mask[Always] | 0.1774497 | 0.019294 | 9.20 | <.0001* |

### Social Contribution

**Parameter Estimates**

| **Term** | **Estimate** | **Std Error** | **t Ratio** | **Prob>\|t\|** |
| --- | --- | --- | --- | --- |
| Intercept | 0.7471872 | 0.025258 | 29.58 | <.0001* |
| Continuous Participant Masking | 0.0235225 | 0.018105 | 1.30 | 0.1941 |
| Target Mask[Always] | 0.3614089 | 0.024567 | 14.71 | <.0001* |
| Class Mask[Always] | -0.098426 | 0.024528 | -4.01 | <.0001* |
| Target Mask[Always]*Class Mask[Always] | 0.0657371 | 0.024506 | 2.68 | 0.0074* |

### Social Acceptance

**Parameter Estimates**

| **Term** | **Estimate** | **Std Error** | **t Ratio** | **Prob>\|t\|** |
| --- | --- | --- | --- | --- |
| Intercept | 0.0136888 | 0.027342 | 0.50 | 0.6167 |
| Continuous Participant Masking | 0.0077476 | 0.019598 | 0.40 | 0.6927 |
| Target Mask[Always] | 0.1210519 | 0.026594 | 4.55 | <.0001* |
| Class Mask[Always] | 0.1195109 | 0.026552 | 4.50 | <.0001* |
| Target Mask[Always]*Class Mask[Always] | 0.2482307 | 0.026528 | 9.36 | <.0001* |

##

#

# Study 3

## Study 3 Inclusion Screening.

Participants were only permitted to access our survey if they self-reported as currently being enrolled in college courses, and participants additional reported each semester -- from Fall, 2020 through Spring, 2023 -- that they were enrolled in at least one in-person college course. Participants also indicated whether they had ever worn a mask to reduce the spread of COVID-19, and were asked to solve a simple word arithmetic problem. Only those participants who successfully responded to each of these questions were invited to complete the full study.

##

## Study 3: Demographics

### Race


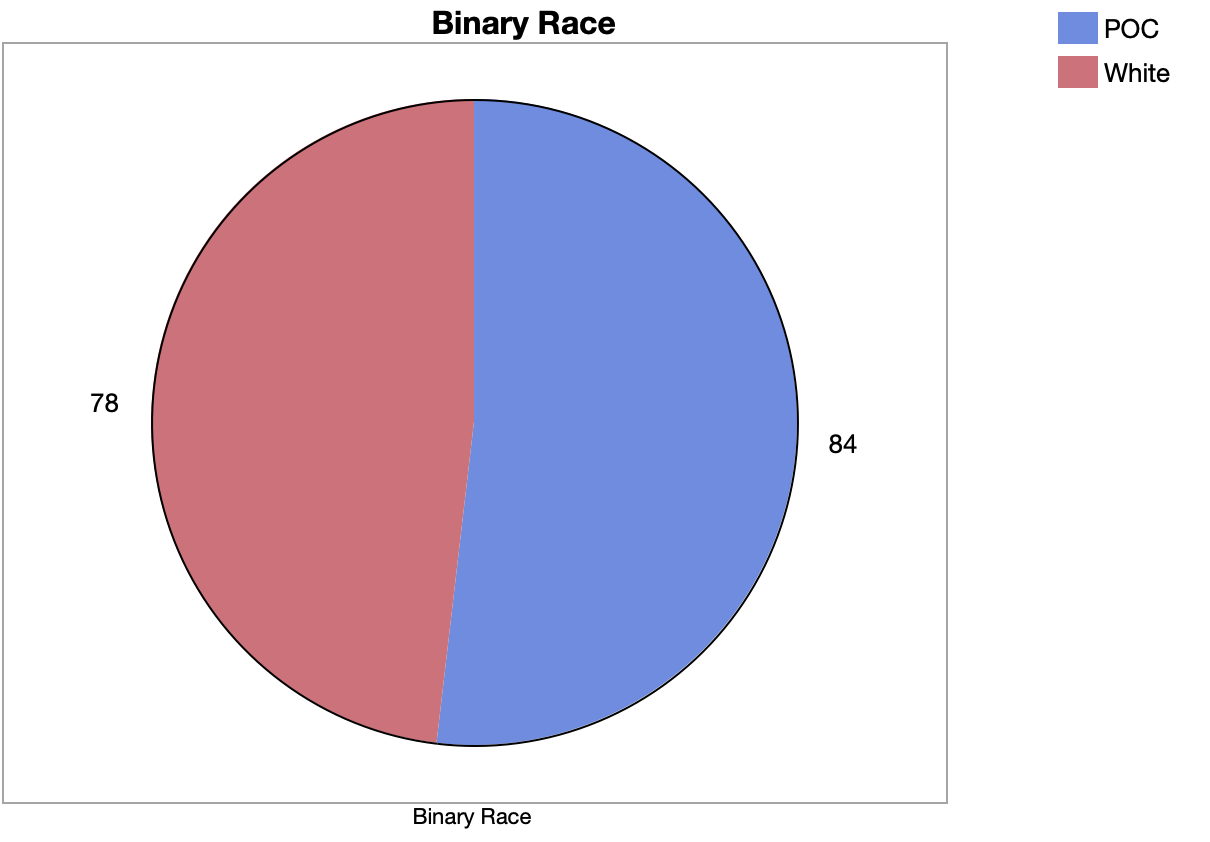


### Minoritized Gender


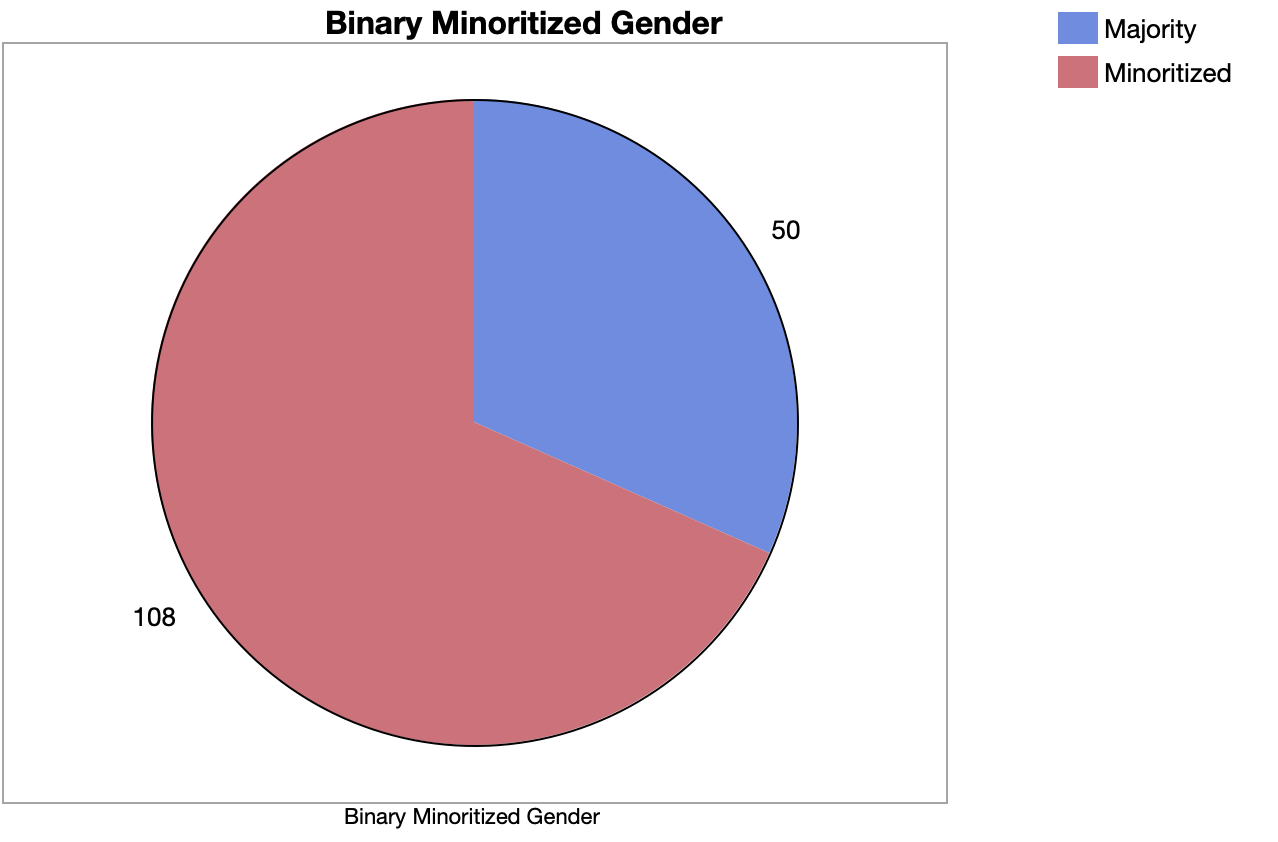


### Participant Immune Status


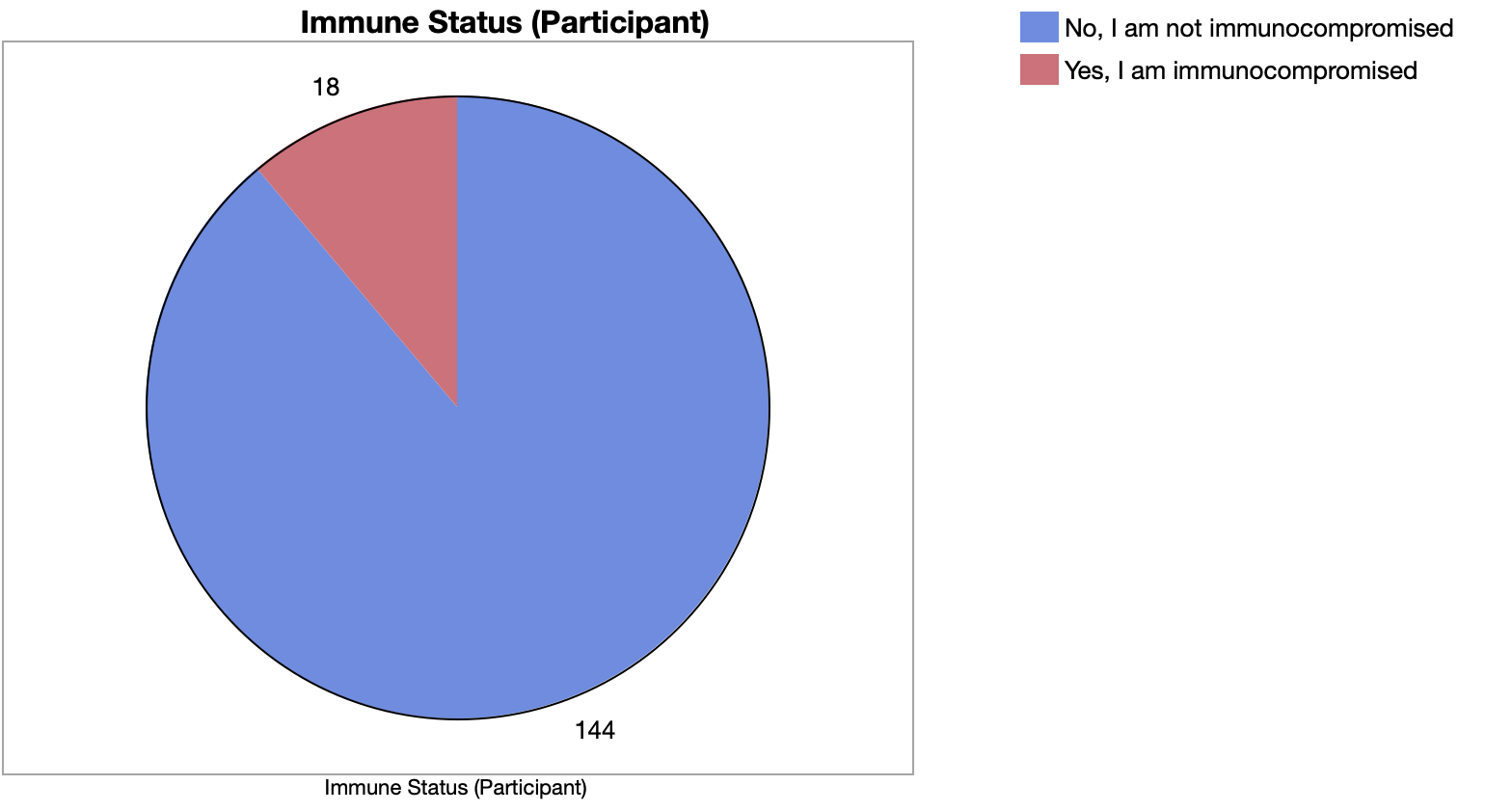


### Participant Masking Behavior


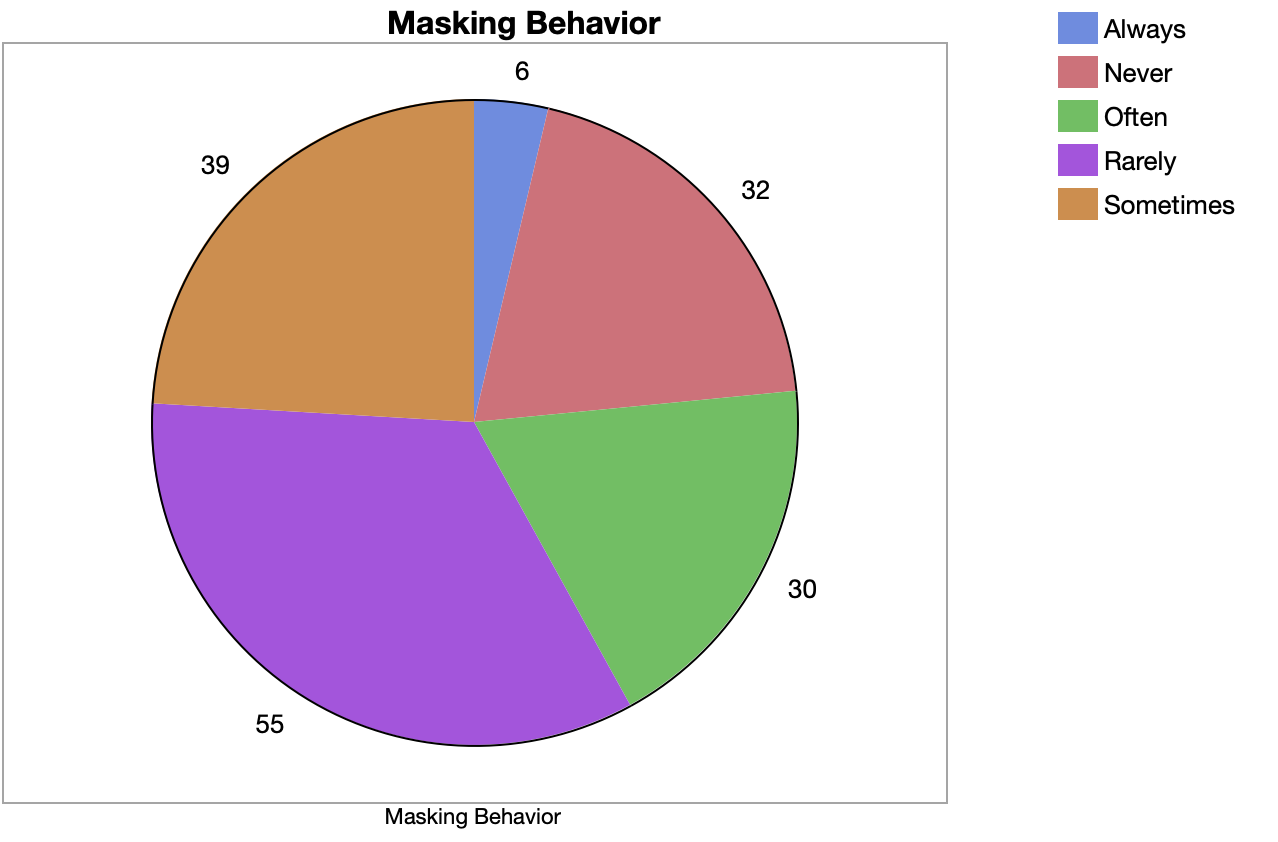


## Study 3: Correlation of Actual and Preferred Masking Behavior

​​Wear Mask = -0.200157 + 0.6110675*Wear Mask Preference

**Summary of Fit**

| RSquare | 0.417714 |
| --- | --- |
| RSquare Adj | 0.414051 |
| Root Mean Square Error | 0.851668 |
| Mean of Response | -0.46584 |
| Observations (or Sum Wgts) | 161 |

**Analysis of Variance**

| **Source** | **DF** | **Sum of Squares** | **Mean Square** | **F Ratio** |
| --- | --- | --- | --- | --- |
| Model | 1 | 82.73323 | 82.7332 | 114.0615 |
| Error | 159 | 115.32889 | 0.7253 | **Prob > F** |
| C. Total | 160 | 198.06211 |  | <.0001* |

**Parameter Estimates**

| **Term** | **Estimate** | **Std Error** | **t Ratio** | **Prob>\|t\|** |
| --- | --- | --- | --- | --- |
| Intercept | -0.200157 | 0.071583 | -2.80 | 0.0058* |
| Wear Mask Preference | 0.6110675 | 0.057216 | 10.68 | <.0001* |

## Study 3: post-hoc analyses of demographics and masking

From prereigstration: “Post-hoc analyses will assess whether demographic factors (e.g., race) interact with masking preference such that individuals from some groups show especially large discrepancies between masking preference and masking behavior.”

There were very few participants who fell into either (a) the quadrant where participants were reporting wearing a mask often/always when they otherwise would prefer to do so rarely/never (*n* = 10) or where (b) participants reported wearing a mask rarely/never when they otherwise would prefer to often/always (*n* = 4). Thus, we have elected not to pursue post-hoc analyses.

### Participant Masking Preference

### Masking Preference vs Behavior


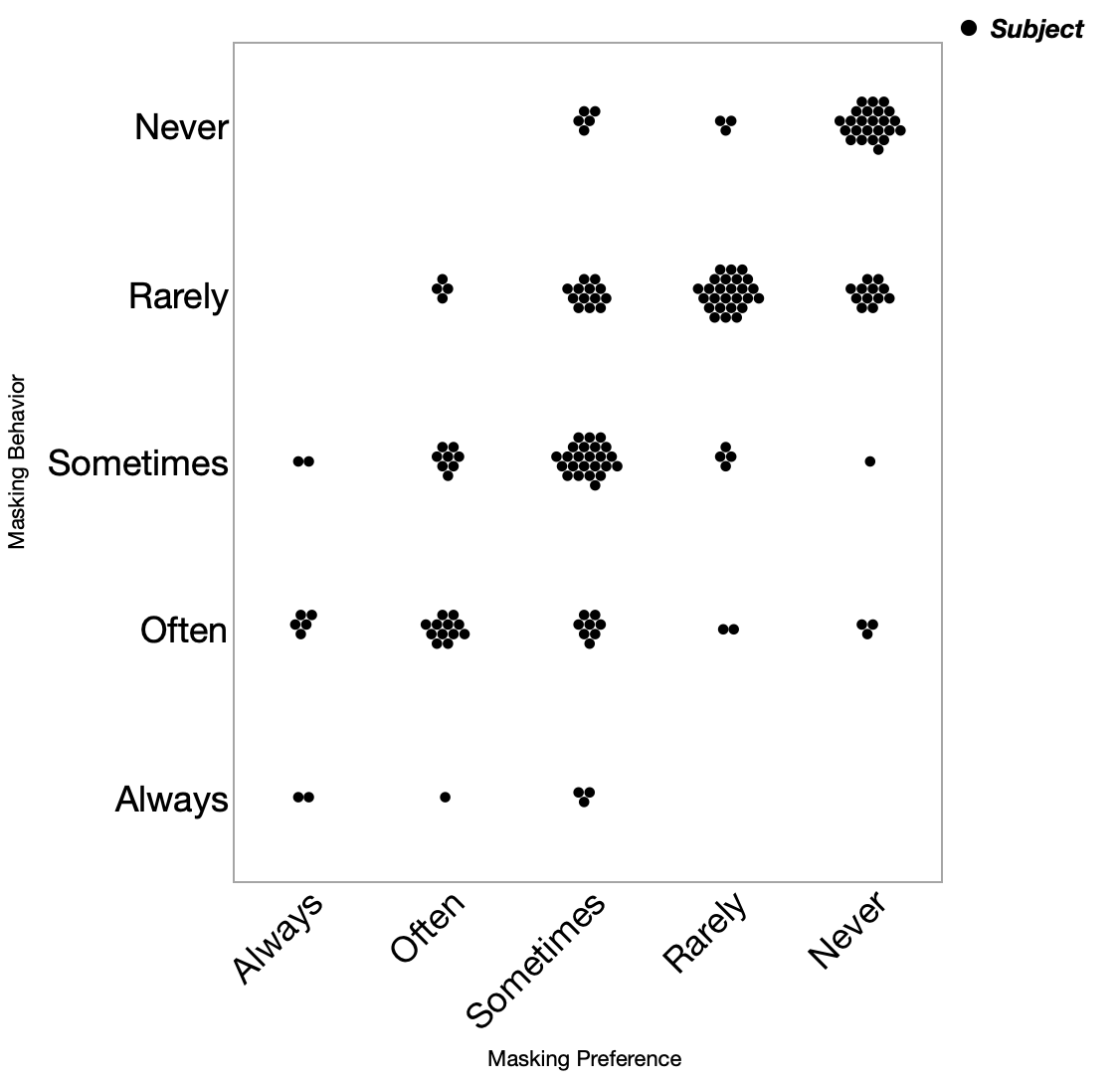


## Study 3: Classroom Enrollment Data

| **I am more likely to want to enroll in/stay in a class if I know that….** | **Percent of participants providing responses above Midpoint** |
| --- | --- |
| the typical masking behavior in the class will match my own | 48% |
| most students in the class will always wear a mask | 36% |
| the professor will always wear a mask | 35% |
| Overall | 60% |

## Study 3: Effects of Demographics on DVs

**Effects of Demographics**.


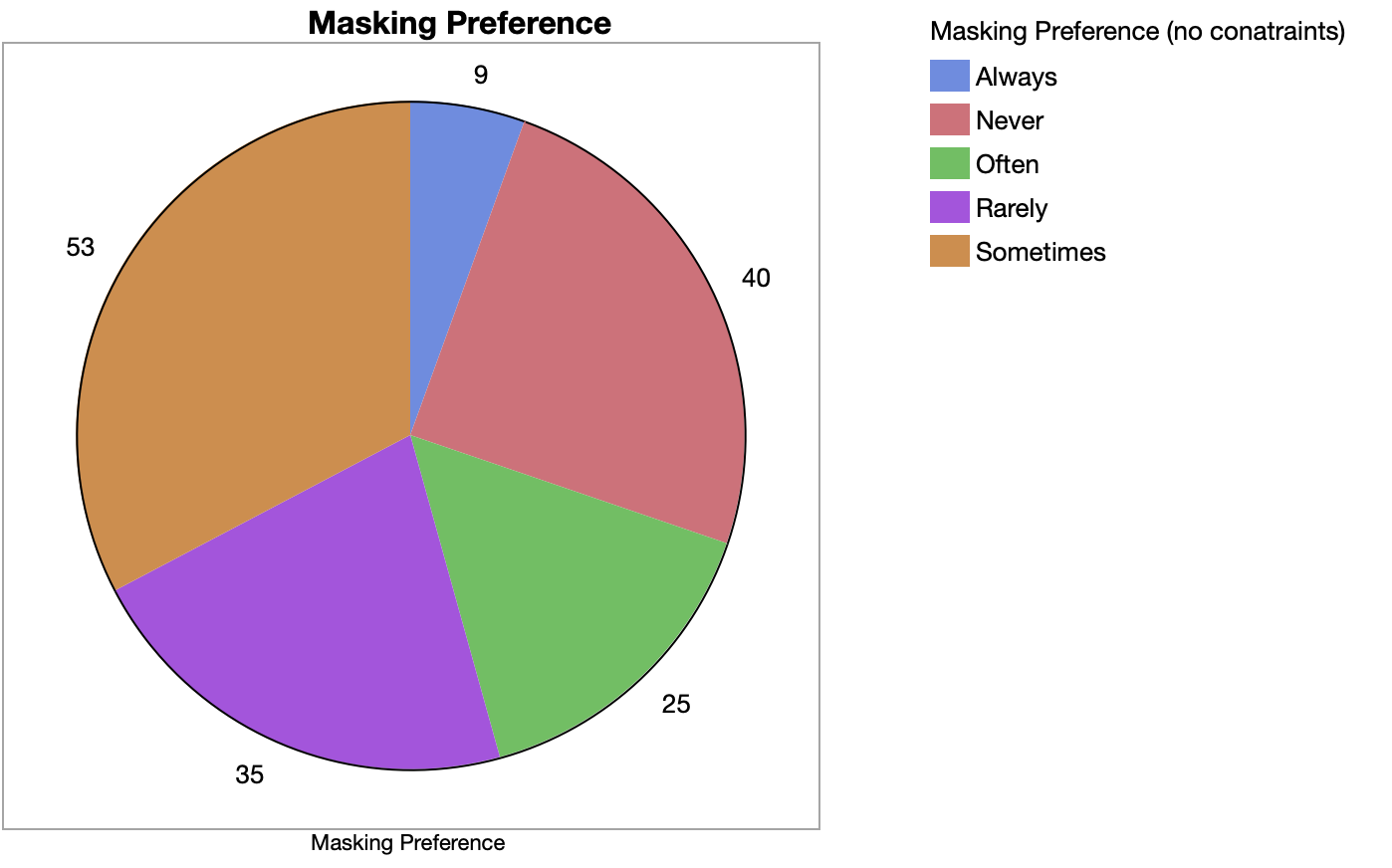

Supplement: S1 Appendix — (DOCX) [file pone.0312392.s001.docx]
