# Supplementary material for: Masking is good, but conforming is better: The consequences of masking non-conformity within the college classroom
Source: PLoS One. 2025 Jan 16;20(1):e0312392. doi: 10.1371/journal.pone.0312392 (PMC11737661; doi:10.1371/journal.pone.0312392)
Supplement: Attachment — Submitted filename: Manuscript PLOS 1 Masking review.docx [file pone.0312392.s002.docx]

Manuscript: PONE-D-24-06722

Masking is good, but conforming is better: The consequences of masking non-conformity within

the college classroom

General:

Timely and interesting manuscript about further exploration of masking behaviors using social and psychological theories. The data is collected from the study participants to give their responses about a hypothetical setting in their own classrooms and results are interpreted based on several “hypotheses” about social and psychological factors. The manuscript presentation is complex because the authors have decided to report three interrelated questions as three separated studies all combined in one manuscript. The difficulty for the audience is the poor delineation and clarity in background, assumptions, hypothetical situations, objectives, and methods for all the three studies. The details (especially background studies, rational and methods) seems mixed up, fragmented, and repetitive in different sections. It seems like there were initially three manuscripts which authors decided to submit as one without making necessary changes. Even though the premise of these studies is interesting, the reader may feel confused or bored (or both) the way this manuscript(s) is written, and results are presented.

Specific Comments:

Useful theoretical information in the introduction. Framing of research questions is appropriate. Rationale of choosing college classrooms setting and broad range of participants is provided. However, the background information, related studies and rationale is incomplete, and it seems that it is provided in different sections later.

The *Current Research* provides overall description of research studies mixed with some methods details. This section does not clearly delineate the distinguishing factors between these studies except studies #1 and 2 were experimental and were aimed at gathering “evidence that real-world masking behavior can be associated with the types of social and academic consequences”, while in study 3# “participants were asked to report their own experiences of other’s responses to their masking choices”.

There are links to the Supplemental online materials in two different locations. Reading the pdf. files in OSF link is crucial, because the assumptions, methods, and outcomes measures are described with more clarity than the manuscript itself.

Even though the details are there, this section seems fragmented and in the presence of individual study objectives, methods, and details, also seems redundant. Therefore, it will be useful for the reader if methods can be written in a more concise and clearer manner, with some sample questions. Our recommendation would be to provide all the background information and rationale in the beginning of the manuscript, followed by a consolidated method, results and discussion sections.

It is not clear whether study participants continuously observed their classrooms over a pre-specified time-period, or they responded based on a one-time observation. (Sorry if this detail was already provided because it is hard to keep up with details spread all over the paper and external supplemental files) Furthermore, all the responses are binary (always wears a mask, never wears a mask) which seems to be somewhat unrealistic. There could be instances where the target (B) chooses to wear a mask for some days when they were not well, but otherwise not 100% adherent. Same is true for the rest of the class or professor. If the authors have a better explanation for choosing a binary in this hypothetical situation, they should justify it with evidence.

The graphs are easy to follow, and the findings are interesting. However, there is hardly anything that surprises us. People like to conform, whether the prevalent culture is a positive or negative behavior. This happens for a fear of perceived social benefits which may overshadow health risks to self or others. Gender effects are also well documented in several studies.

So, making a culture of masking in the classroom will have the most impact on individuals’ behavior and their perception of being fit for the classroom environment.

Please add a description of limitations of this study in terms of study assumptions, study design, and participant recruitment.
