# Supplementary material for: Masking is good, but conforming is better: The consequences of masking non-conformity within the college classroom
Source: PLoS One. 2025 Jan 16;20(1):e0312392. doi: 10.1371/journal.pone.0312392 (PMC11737661; doi:10.1371/journal.pone.0312392)
Supplement: Attachment — Submitted filename: Response to Reviewers (1).docx [file pone.0312392.s003.docx]

Dear Dr. Clark,

Thank you for the opportunity to revise and resubmit our manuscript, entitled “Masking is good, but conforming is better: The consequences of masking non-conformity within the college classroom” (PONE-D-24-06722). My co-authors and I are delighted that the paper will be further considered for publication in *PLOS ONE*. We wish to clarify that the funders had no role in study design, data collection and analysis, decision to publish, or preparation of the manuscript.

We have substantially revised the manuscript to address the excellent comments offered by you and the two anonymous reviewers. Your collective feedback allowed us to significantly improve the paper, and we are grateful for your insights. A detailed list of our responses to the comments follows below the signature in this letter. Among many other improvements, you will see that in keeping with suggestions, we have:

1. Worked to clarify the relations between our studies at both the theoretical and methodological level. To this end, we have added a “Research Overview” section, added a table specifying the relations between our studies, added a table summarizing our scales across studies, and have revised the Method section for each study to increase clarity and reduce redundancy.
2. Worked to situate our research more obviously within the social psychology literature. To this end, we have revised our Abstract, restructured our Introduction, and added a wealth of literature that more clearly establishes the connection between our study and previous work. As part of the revision process, we also more clearly outlined our hypotheses.
3. Moved tables from the supplement to the main text in order to streamline the reading experience and clarify our reporting.

Thank you again for your consideration. We are grateful for the enthusiasm that you and the reviewers expressed for the current research (in addition to the valuable constructive feedback), as we believe that this work examines an important yet under-explored topic. Please do not hesitate to contact me if there is anything that I can clarify, or if you feel that there are additional points that should be addressed. We sincerely hope you will agree that this significantly revised version of the manuscript is worthy of publication in *PLOS ONE,* and we look forward to receiving your feedback.

Sincerely,

The authors

**Editorial Comments**

1. The topic of masking is one that became critical and may again in the future so it is helpful to understand this issue. However, the reviewers had a difficult time following your 3 different studies. They have provided you with feedback on ways to help you clarify your work. Be respond carefully to their concerns.

**We thank the reviewers and the editor for their helpful feedback. We are glad that both the editor and the reviewers see the promise and value in this work, and have addressed all concerns in detail (with a particular eye to adding clarity across the 3 studies). We believe that the resulting paper is better structured, and as a result, substantially strengthened. Please see below for detailed responses to all of the reviewers’ comments.**

2. Please include the following items when submitting your revised manuscript:

- A rebuttal letter that responds to each point raised by the academic editor and reviewer(s). You should upload this letter as a separate file labeled 'Response to Reviewers'.
- A marked-up copy of your manuscript that highlights changes made to the original version. You should upload this as a separate file labeled 'Revised Manuscript with Track Changes'.
- An unmarked version of your revised paper without tracked changes. You should upload this as a separate file labeled 'Manuscript'.

**We are including the rebuttal letter, a marked-up copy of our manuscript, and a revised manuscript without markings.**

3. Please ensure that your manuscript meets PLOS ONE's style requirements, including those for file naming. The PLOS ONE style templates can be found at
   <https://journals.plos.org/plosone/s/file?id=wjVg/PLOSOne_formatting_sample_main_body.pdf> and
   <https://journals.plos.org/plosone/s/file?id=ba62/PLOSOne_formatting_sample_title_authors_affiliations.pdf>

**We have double checked the formatting requirements and are in compliance. We have named our files as requested above. The one exception is to the requirement for reference ordering. Because of the time investment involved in formatting the references to fit PLoS’s specifications, we would prefer to put all references in journal-compliant format if/when it becomes evident that the article is likely to be accepted. At that time, we will also update all figures to high resolution.**

4. We note that the grant information you provided in the ‘Funding Information’ and ‘Financial Disclosure’ sections do not match. When you resubmit, please ensure that you provide the correct grant numbers for the awards you received for your study in the ‘Funding Information’ section. Thank you for stating the following financial disclosure:
   "Funding from Skidmore College was awarded to JS in support of this project. "

**We believe that our funding information matches. Our funding was internal institutional funding from Skidmore College awarded to JS in the form of a “Faculty Research Initiative Grant”, and as such, there was no award number. If there is a different way to report this, we are happy to make whatever changes are required to meet compliance standards.**

5. Please state what role the funders took in the study. If the funders had no role, please state: "The funders had no role in study design, data collection and analysis, decision to publish, or preparation of the manuscript." If this statement is not correct you must amend it as needed. Please include this amended Role of Funder statement in your cover letter; we will change the online submission form on your behalf.

**The funders had no role in study design, data collection and analysis, decision to publish, or preparation of the manuscript. We have clarified this in the response letter above.**

6. Please include your full ethics statement in the ‘Methods’ section of your manuscript file. In your statement, please include the full name of the IRB or ethics committee who approved or waived your study, as well as whether or not you obtained informed written or verbal consent. If consent was waived for your study, please include this information in your statement as well.

**We have moved our Ethics Statement to the Study Overview, since it applied to each of our three studies. We are also happy to write three identical ethics statements (one for each study) if that is preferred/required.**

7. Please include a copy of Table 3 which you refer to in your text on page 36.

**We have updated and fixed all table references.**

**Reviewer #1**

1. Thank you for the opportunity to review this manuscript. General: Timely and interesting manuscript about further exploration of masking behaviors using social and psychological theories. The data is collected from the study participants to give their responses about a hypothetical setting in their own classrooms and results are interpreted based on several “hypotheses” about social and psychological factors.

**We thank the reviewer for their thoughtful read of the paper, and for their helpful suggestions, which we respond to below.**

2. The manuscript presentation is complex because the authors have decided to report three interrelated questions as three separated studies all combined in one manuscript. The difficulty for the audience is the poor delineation and clarity in background, assumptions, hypothetical situations, objectives, and methods for all the three studies. The details (especially background studies, rational and methods) seems mixed up, fragmented, and repetitive in different sections. It seems like there were initially three manuscripts which authors decided to submit as one without making necessary changes. Even though the premise of these studies is interesting, the reader may feel confused or bored (or both) the way this manuscript(s) is written, and results are presented.

**We are glad that despite the complexity introduced by reporting on three separate studies, the reviewer nevertheless found the premise of the studies to be interesting. Many of our revisions were focused on streamlining the structure of the paper. To this end, we made several changes, outlined below.**

**First, we have removed the “Current Research” section from the previous Introduction, and have instead added a “Research Overview” section prior to Study 1, and distinct from the Introduction. This section explicitly explains the goals of each study, and their similarities and differences. We have created a table (Table 1) to highlight the similarities and differences between the studies, and have more clearly motivated each study within the “Research Overview” section. We hope that these changes will make it easier for future readers to see the similarities and differences across studies, and that these changes will also make it easier for readers who may only be interested in a subset of our research to locate the stud(ies) that are most useful to them. Finally, while we have elected to keep the mini-introductions that currently introduce the elements that are unique to each study, we have foreshadowed and explained our choice to do so in the main text.**

**Second, in response to comments from both reviewers, we moved a table that had provided information about our scales from the Supplemental Online Materials to the main text. In doing so, we hope that it will be easier for readers to follow which measures showed up in which studies. This table (Table 2) describes each scale, which studies each scale appeared in, the source(s) for the scales, the reliability scores for the scales, and the poles for each scale. In addition, we provide sample items from each scale within the table. Critically, inserting this table into the main text has allowed us to greatly shorten the Method section for Studies 2-3, reducing redundancy. We also suspect that it will substantially increase readability, both for readers who wish to read the entirety of the manuscript and for readers who may be particularly interested in a subset of the studies that we present.**

3. Useful theoretical information in the introduction. Framing of research questions is appropriate. Rationale of choosing college classrooms setting and broad range of participants is provided. However, the background information, related studies and rationale is incomplete, and it seems that it is provided in different sections later.

**We thank the reviewer for noting the strengths of our previous framing and theoretical information in the introduction. We agree with both reviewers that there were further opportunities to provide a richer theoretical background in the introduction. To this end, we have made substantial edits to the introduction, more clearly locating our primary research questions (e.g., the perceived impacts of masking behavior and masking conformity on students) within the social psychological and health psychological literatures. As a result, the Introduction now provides a richer theoretical framing for all three of our Studies.**

**We continue to provide study-specific rationale at the start of each study to avoid cluttering the general introduction. We suspect doing so will also make this document easier to read for individuals who may be most interested in reading just a subset of our studies.**

4. The Current Research provides overall description of research studies mixed with some methods details. This section does not clearly delineate the distinguishing factors between these studies except studies #1 and 2 were experimental and were aimed at gathering “evidence that real-world masking behavior can be associated with the types of social and academic consequences”, while in study 3# “participants were asked to report their own experiences of other’s responses to their masking choices”.

**We now see that the previous “Current Research” section of the Introduction did not adequately clarify the similarities and differences between the three studies, nor did it improve the readability of the text. As we note above, we have completely restructured this section. We have made it its own section (instead of placing it at the end of the Intro), we have added a table to clarify the differences and similarities in methods (see above), and have worked to clarify the differences in research questions asked across studies.**

5. There are links to the Supplemental online materials in two different locations. Reading the pdf. files in OSF link is crucial, because the assumptions, methods, and outcomes measures are described with more clarity than the manuscript itself. Even though the details are there, this section seems fragmented and in the presence of individual study objectives, methods, and details, also seems redundant. Therefore, it will be useful for the reader if methods can be written in a more concise and clearer manner, with some sample questions. Our recommendation would be to provide all the background information and rationale in the beginning of the manuscript, followed by a consolidated method, results and discussion sections.

**Thank you for this recommendation. Most critically, we have moved some of the materials from the supplement to the main text, allowing us to foreground the sample questions and methods. The online Supplement now *only* contains supplemental analyses that are not germane to the main findings or arguments in the text, and does not contain any supplemental information about methods. We note this clearly in-text throughout the manuscript.**

**In addition, we have updated our links so that our OSF page (where all study materials are provided in full) are provided when we are describing how researchers can access full study materials should they wish to directly replicate our work using exactly the materials that we used.**

**All links have been updated.**

6. It is not clear whether study participants continuously observed their classrooms over a pre-specified time-period, or they responded based on a one-time observation.

**For Studies 1-2, participants read about a classroom scenario a single time. Because Study 3 asked for information about the participant’s own experiences with masking, we suspect it is likely that their responses were informed by many observations. However, the free-response prompt requested that participants “recall a time when…”, predisposing them to write about a single event.**

7. Furthermore, all the responses are binary (always wears a mask, never wears a mask) which seems to be somewhat unrealistic. There could be instances where the target (B) chooses to wear a mask for some days when they were not well, but otherwise not 100% adherent. Same is true for the rest of the class or professor. If the authors have a better explanation for choosing a binary in this hypothetical situation, they should justify it with evidence.

**We agree that in the real world, masking behavior is not always binary, even though a sizeable subset of our participant pool reported their *own* masking in binary terms. Specifically, [38.9%_Study1_; 39.8%_Study2_ ; 22.3%_Study3_] of our participants described their masking behavior in binary (“always” or “never”) terms, instead of selecting from our more nuanced options like “rarely”, “sometimes”, “often”. This is consistent with polling data cited in our papers, which showed that 58-63% of adults report their masking behavior in binary terms. So, in the context in which our data were collected, binary masking behavior likely did not seem unusual.**

**In Studies 1-2, we *manipulated* binary masking behavior in order to maximize the likelihood of detecting possible effects of conformity. In Study 3, we used methods that were more sensitive to non-binary masking behavior. Specifically, instead of responding about binary masking behavior, participants were asked to provide a free-response about “a time when they [were/were not] wearing a mask while most people in their environment [were/were not] wearing a mask”. Here, we explicitly measured reactions to circumstances that went beyond always/never masking. Further, in Study 3, we introduced scales that measured the impact of masking on students’ experiences -- these scales referred to “masking behavior” instead of specifying binary masking. Critically, despite large methodological differences for Study 3 vs. the earlier studies (i.e. use of free response, asking about scenarios that went beyond an always/never masking binary) we found a consistent pattern of responses: strong masking conformity effects.**

8. The graphs are easy to follow, and the findings are interesting.

**Thank you!**

9. However, there is hardly anything that surprises us. People like to conform, whether the prevalent culture is a positive or negative behavior. This happens for a fear of perceived social benefits which may overshadow health risks to self or others. Gender effects are also well documented in several studies. So, making a culture of masking in the classroom will have the most impact on individuals’ behavior and their perception of being fit for the classroom environment.

**We have responded to this concern in two primary ways.**

**First, this comment -- along with comments from Reviewer 2 -- made us realize that we had failed to adequately convey the numerous plausible (and theoretically-motivated!) outcomes for our studies. We have substantially edited the introduction to better set up these different theoretical possibilities. Indeed, while conformity effects are potentially predicted, so are many other types of effects, each motivated by the existing literature. For example, we might expect simple effects of masking, based on the large extant literature demonstrating valenced judgments based on individuals’ clothing and self-presentation decisions. A second possibility -- based both on the backlash literature and on the literature on self-presentation -- is that effects of masking might appear *only* (or especially) for certain social groups. A final possibility (not mutually exclusive with the first two) is the one that is best supported by our data: that masking-conformity may play the strongest role in shaping perceptions. Our studies were designed to test each of these possibilities, and found the strongest support for the last of them.**

**Second, we highlight in the Discussion that -- while our overall findings are consistent with previous conformity literature -- there is substantial applied value to demonstrating that (a) conformity effects *do* arise from masking behavior and (b) that these effects impact both the perceptions of students [Studies 1 and 2] and students’ self-reported behaviors [Study 3]. Indeed, we suspect that many readers of this manuscript will be primarily interested in these data for their applied value.**

**Finally, while we do not dwell on this in the main text of the manuscript, it is worth noting that a quick examination of Figure 2 reveals that the manifestation of masking effects *does* differ across scales. These data suggest to us that the types of conformity effects that we robustly demonstrate for ‘perceived classroom fit’ are not the only types of effects of masking that we can detect.**

10. Please add a description of limitations of this study in terms of study assumptions, study design, and participant recruitment.

**We have restructured our limitations to include three subheaders: Limitations to Participant Recruitment, Limitations to Study Design, and Other Limitations to Study Interpretation.**

**Reviewer #2**

11. Thank you for the opportunity to review this manuscript. The authors present a series of three studies aimed at exploring the impact of masking and not masking on social and academic judgments of students within the college classroom. The series has coherence and use mixed-methods to have a wide picture of the phenomenon. This reviewer appreciates the authors' effort to collect such an amount of data on a topic that nowadays is fully pertinent.

**We sincerely appreciate this positive feedback! As you can imagine, collecting these data was a big lift, and we are glad that the methodological choices that we made improved the depth of our conclusions.**

12. I have missed, both in the Introduction and Discussion, more reference to the vast literature on peer pressure, social acceptance, majority and minority influence, and related topics. I understand the irruption of COVID-19 has motivated research on topics that look new but that, indeed, are not. Articles on masking are dated from 2020. But masking is just another example of behavior that is subjected to social pressure, same as alcohol use and abuse, gender-consistent behaviors, and the like. As masking is not a "new" behavior, IMHO research on masking cannot focus on showing a picture of the phenomenon but should also explain such phenomenos according to one of more of the theories that have tradicionally addressed social pressure and majority and minority influence.

**We appreciate these important points, and have meaningfully revised the manuscript to better ground the current working in the existing literature. More specifically, throughout the introduction, we now provide an in-depth discussion of several relevant theoretical perspectives and bodies of literature pertaining to group influence, and focus on presenting our work within these theoretical and empirical contexts.**

**As this reviewer clearly articulated, masking behavior is a timely, relevant, and potentially consequential behavior, but/and, it is subject to established social processes impacting many types of social behaviors. We hope that in the current manuscript, we have more clearly articulated the ways in which our work on the impact of social pressures on masking (one particular, more novel behavior) fits into social psychology’s broader history of studying the impact of social pressure on many different types of behaviors in context.**

13. Authors are heavily relying on data collected with ad-hoc measures consisting on a few items. They report the alpha, that is good, but sometimes it is barely acceptable (around .70). The fact that data are not collected through validated measures may pose a problem and should be, at the least, acknowledged as a limitation.

**We have clarified our use of scales in the manuscript, and, per Reviewer 1’s suggestion, have moved a Table from the Supplement to the Main text (Table 2). This table specifies the scale name, number of items, examples of items, source of the scale, alpha, and interpretation of each scale. We want to highlight that our scales contained a mix of custom materials (e.g., the Classroom Fit scale) and previously validated scales.**

**In general, we note that α was relatively high. The reviewer is correct that in one of our studies -- for only one of our scales -- the alpha was as low as .71. Critically, for “the early stages of research” values as low as .70 are sufficient (Nunnally, 1978 via Lance, Butts, & Michel, 2006). We feel that these criteria are appropriate for Study 1, which -- by virtue of its novel focus on COVID-19 and face-masking -- was the early stages of research. Critically, by Study 3, we had iterated on this scale (combining it with other items to create the Mask Impact Scale), arriving at a far stronger measure with α = .90.**

**In addition to clarifying this in the main text, we have added a clear statement in the Discussion of the potential limitations and challenges of developing novel scales in response to novel circumstances (e.g., rapidly changing masking norms).**
